# Synthesis of fluorinated maltose derivatives for monitoring protein interaction by ^19^F NMR

**DOI:** 10.3762/bjoc.8.51

**Published:** 2012-03-27

**Authors:** Michaela Braitsch, Hanspeter Kählig, Georg Kontaxis, Michael Fischer, Toshinari Kawada, Robert Konrat, Walther Schmid

**Affiliations:** 1 Department of Organic Chemistry, University of Vienna, Währinger Strasse 38, A-1090 Vienna, Austria; 2 Department of Structural and Computational Biology, Max F. Perutz Laboratories, University of Vienna, Campus Vienna Biocenter 5, A-1030 Vienna, Austria; 3 Graduate School of Life and Environmental Sciences, Kyoto Prefectural University, Sakyo-Ku, Kyoto 606-8522, Japan

**Keywords:** fluorination, ^19^F NMR, maltose-binding protein (MBP), maltose derivatives, protein interaction

## Abstract

A novel reporter system, which is applicable to the ^19^F NMR investigation of protein interactions, is presented. This approach uses 2-F-labeled maltose as a spy ligand to indirectly probe protein–ligand or protein–protein interactions of proteins fused or tagged to the maltose-binding protein (MBP). The key feature is the simultaneous NMR observation of both ^19^F NMR signals of gluco/manno-type-2-F-maltose-isomers; one isomer (α-gluco-type) binds to MBP and senses the protein interaction, and the nonbinding isomers (β-gluco- and/or α/β-manno-type) are utilized as internal references. Moreover, this reporter system was used for relative affinity studies of fluorinated and nonfluorinated carbohydrates to the maltose-binding protein, which were found to be in perfect agreement with published X-ray data. The results of the NMR competition experiments together with the established correlation between ^19^F chemical shift data and molecular interaction patterns, suggest valuable applications for studies of protein–ligand interaction interfaces.

## Introduction

In recent years, we have witnessed significant improvements in NMR spectroscopy, especially as a powerful tool for studying protein–ligand and protein–protein interactions [1–2]. Based on tremendous gains in sensitivity due to high-field spectrometers and cryogenic-probe technology, unprecedented structural and functional information can be obtained on biologically important protein–ligand systems and protein complexes [2]. To overcome the well-known and inherent problem of molecular weight limitation of current NMR spectroscopy, which renders direct protein observation of the interaction partners infeasible, an indirect observation technique for the detection of protein interactions was recently established [3]. It utilizes the relaxation properties of a small-molecular-weight reporter ligand that reversibly binds to a ligand binding domain, which in turn is fused to the interacting protein of interest. Subsequent protein–protein interaction leads to an additional increase of the molecular weight of the complex and can efficiently be probed by following the NMR relaxation changes of the ligand (e.g., selective T_1_ or T_2_, which reflect the effective molecular weight). Due to this indirect detection scheme no isotope labeling of the protein interaction partners is required and consumption of protein material is reduced.

The concept presented here relies on the development of an indirect ^19^F-detected NMR reporter system with possibilities for internal control for the study of protein-binding events. The benefits of fluorine (^19^F) NMR detection for ligand-based NMR screening applications as well as for ^19^F magnetic resonance imaging (MRI) have been convincingly demonstrated in the past [4–11]. The usage of the fluorine NMR alleviates most of the problems encountered with ^1^H observation, such as signal overlap and problems with the dynamic range. Additionally, the ^19^F nucleus with 100% natural abundance and a magnetogyric ratio comparable to ^1^H is highly sensitive and, due to its large chemical shift anisotropy (CSA), very responsive to changes of molecular weight that accompany the binding events.

We thus anticipate ^19^F detection to be a general and versatile probe for indirect NMR studies of protein-binding and interaction events. Biological systems often require sophisticated buffer systems for stabilization and solubility, thus leading to severe spectral overlap and problems with the dynamic range (e.g., intense buffer and solvent peaks). These drawbacks are particularly present in the case of membrane-bound (or attached) proteins, in which additional peaks originate from membrane lipids and raise severe technical problems. However, indirect detection techniques should always be cross-checked with reference experiments and suitable controls, to demonstrate selectivity of binding and to exclude systematic errors (e.g., nonspecific binding or aggregation, and/or viscosity changes due to increased protein concentration). Ideally, the system of choice would thus be a mixture of reporter ligands consisting of one ^19^F-labeled reporter ligand and another chemically similar (also ^19^F-labeled) reference compound lacking the affinity to the ligand binding domain.

Here we describe the possibility of monitoring protein interactions by ^19^F NMR, known as fluorine chemical-shift anisotropy and exchange for screening (FAXS) [5–7], with internal control by using 2-F labeled maltose as a reporter system. The rationale for choosing maltose lies in the fact that maltodextrin/maltose-binding protein (MBP) is a generally applicable protein fusion tag with beneficial solution properties and therefore widely used in molecular biology [12–13].

**Figure 1:**
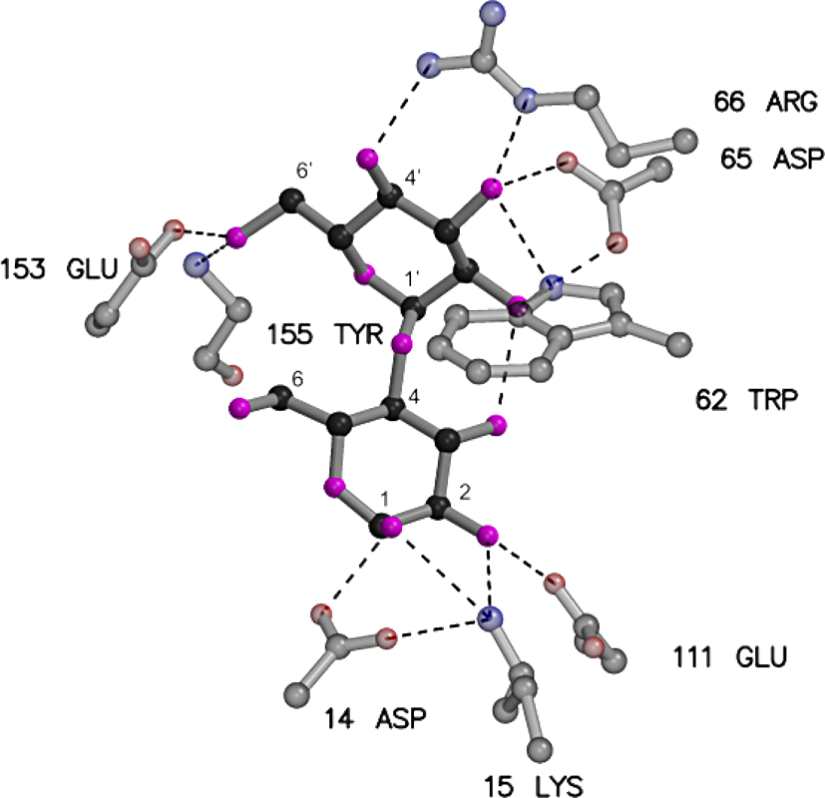
Schematic diagram of the network of hydrogen bonds in the binding pocket of the complex between MBP and maltose (PDB ID code 1ANF); hydrogen bonds are shown as dashed lines.

MBP belongs to the family of periplasmic binding proteins, which are involved in active transport processes of small molecules into gram-negative bacteria through their function as an initial high-affinity binding component; furthermore, these proteins participate as sensors for signaling during chemotaxis [14]. MBP binds maltodextrin and linear oligosaccharides of up to eight α(1→4)-linked glucose (Glc) units with micromolar affinities [15–16]. X-ray structural data (PDB ID codes 1-DMB and 1ANF) demonstrated that the MBP (370 residues, *M*_r_ = 41 kDa) consists of two globular domains joined by a hinge-bending region, in which the ligand binding site is located in a cleft between the two domains. MBP exists in two different conformations: The ligand-free “open” form, exposing the binding site, and in the presence of a ligand, the “closed” form, trapping the ligand to provide contacts from both domains [17–19]. The number of protein–sugar hydrogen bonds associated with maltose and MBP is 12, excluding those with water and between glucose units. The reducing glucose unit (g_1_) makes about twice as many direct hydrogen bonds with MBP as the nonreducing glucose unit (g_2_) does (Figure 1). But there is some evidence for the importance of hydrogen bonds and van der Waals interactions for the oligosaccharide binding as well [20–22].

**Figure 2:**
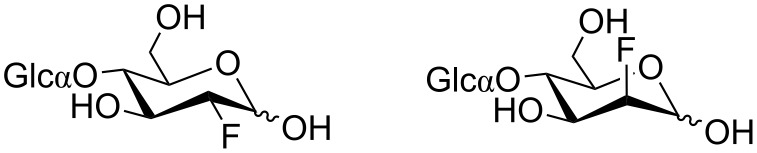
2-^19^F-Maltose reporter system: Nonstereoselective fluorine labeling at the 2-position of maltose leads to a 2/1 mixture of two epimeric forms [left: gluco-type; right: manno-type]. Only the gluco-type isomer of 2-deoxy-2-fluoro-maltose retains the affinity to the maltose-binding protein (MBP).

Specifically, the 2-OH and the 2′-OH moieties are involved in an intricate hydrogen bonding network including the carboxy group of Glu111 and Asp65 and the amino group of Lys15 and Trp62, respectively. We thus decided to synthesize 2-^19^F-labeled maltose. By replacing the OH group by fluorine and modifying the stereochemistry at position 2, different binding affinities of the anomeric mixture of the two resulting diastereomers were expected (Figure 2). The gluco-type 2-F-maltose, in which the fluorine atom occupies the equatorial position of g_1_ of maltose, should display comparable binding affinities to maltose itself, whereas the manno-type 2-F-maltose was expected to lose its affinity due to the axial orientation of the fluorine atom.

This ^19^F-labeled reporter experiment (FAXS) [5–7] was additionally used to measure the relative binding affinities of various fluorinated and nonfluorinated maltose derivatives to MBP in competitive titration experiments. The incorporation of fluorine in different positions into maltose allows fine tuning of the carbohydrate affinities to the maltose-binding protein.

## Results and Discussion

### Syntheses

**Scheme 1:**
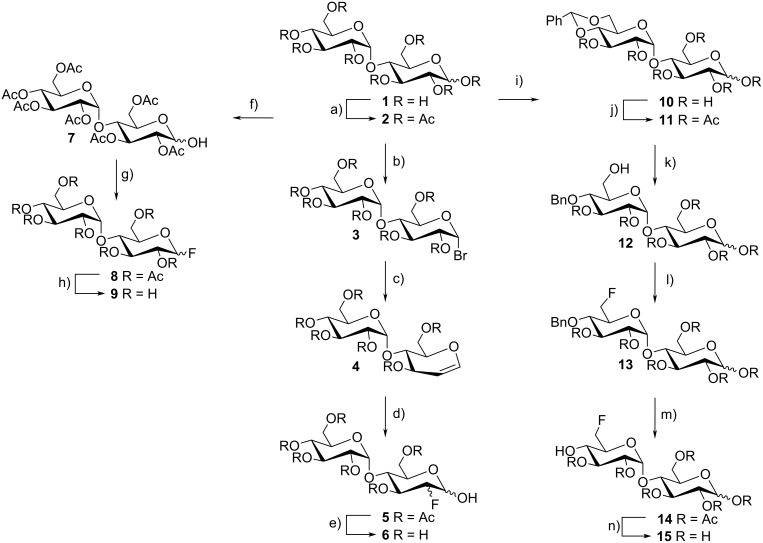
Syntheses of maltose derivatives; reagents and conditions: (a) Ac_2_O, Pyr, 97%; (b) HBr, AcOH, 99%; (c) Zn, *N*-methylimidazole, ethyl acetate, 74%; (d) Selectfluor^®^, CH_3_NO_2_, 40%; (e) NaOMe, MeOH, 99%; (f) NH_2_NH_2_·HOAc, DMF, 94%; (g) DAST, CH_2_Cl_2_, 89%; (h) NaOMe, MeOH, 99%; (i) α,α-dimethoxytoluene, *p*-TosOH, DMF, 79%; (j) Ac_2_O, Pyr, 93%; (k) BH_3_·THF, Bu_2_BOTf, THF 56%; (l) microwave reaction, DAST, collidine, CH_2_Cl_2_, 79%; (m) Pd/C, H_2_, ethylacetate, 64%; (n) NaOMe, MeOH, 75%.

The synthesis of the 2-F-maltose reporter system was performed following a modified protocol developed by Dax et al. [23–24]. Starting from maltose (**1**), disaccharide α-bromide **3** was obtained in excellent yield by a standard acetylation procedure and subsequent treatment with hydrobromic acid in glacial acetic acid (Scheme 1) [25]. Treatment of bromide **3** with Zn and *N*-methylimidazole [26] afforded the protected maltal derivative **4**, which was transformed to the target compounds by utilizing Selectfluor^®^ as a fluorinating agent [23,27–28] in a nitromethane solution. The mixture of anomeric 2-fluoro derivatives **5** with gluco- and manno-type stereochemistry was analyzed by ^19^F NMR, thus showing a gluco (α/β = 1/1) to manno (α/β = 2/1) ratio of 2/1. Final deprotection with sodium methoxide yielded the deprotected fluoro-derivatives **6**.

**Scheme 2:**
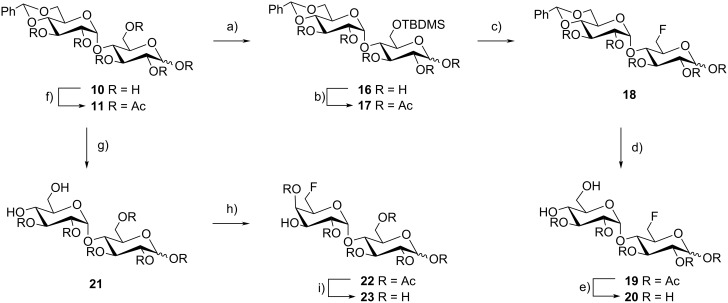
Synthesis of the maltose- and galacto-type derivatives; reagents and conditions: (a) TBDMS-Cl, imidazole, DMF, 43%; (b) Ac_2_O, Pyr, quant.; (c) Deoxofluor, CH_2_Cl_2_, 17%; (d) conc. AcOH, 73%; (e) NaOMe, MeOH, 40%, (f) Ac_2_O, Pyr, 93%; (g) conc. AcOH, 76%; (h) DAST, collidine, CH_2_Cl_2_, 30%; (i) NaOMe; MeOH, quant.

Maltosyl fluoride **9** was obtained by deprotection of the anomeric acetyl group of compound **2** with hydrazine acetate [29] yielding derivative **7**, followed by nucleophilic fluorination with DAST [30–31] generating the diasteriomeric mixture **8**. The α-anomer was isolated by HPLC and subsequent Zemplén saponification of the remaining acetate protecting groups yielded the α-maltosyl fluoride **9**. However, the β-maltosyl fluoride turned out to be rather unstable. Decomposition of the unprotected fluorinated sugar to maltose and hydrofluoric acid started immediately in D_2_O-solution. Therefore only the α-maltosyl fluoride was used for the binding studies. The regioselective reductive ring opening of benzylidene acetals in the maltose derivative **11** was performed with a complex of BH_3_/Bu_2_BOTf at −70 °C [32–33]. Fluorination with DAST [34–35] was performed in a sealed tube for 1 h at 80 °C under microwave conditions. The deprotection of the benzyl group was achieved with Pd/C [36], followed by a Zemplén saponification to obtain product **15**. Starting from 4′,6′-*O*-benzylidene maltose **10** [37], the primary alcohol was protected as *tert*-butyldimethylsilyl ether followed by standard peracetylation (Scheme 2). Treatment of the silyl protecting group with an excess of Deoxofluor [38] yielded the 6-F-maltose derivatives **18**. Final deprotection with acetic acid [37,39] and sodium methoxide yielded compound **20**.

The synthesis of the galacto-type derivative **23** started from peracetylated benzylidene maltose **11** [37]. Deprotection [37] with acetic acid followed by microwave fluorination with DAST [34–35] yielded a mixture of fluorinated disaccharides: The desired product **22** [39] was isolated by column chromatography and Zemplén deprotection yielded derivative **23**.

### Binding studies using the 2-F-maltose reporter system

**Figure 3:**
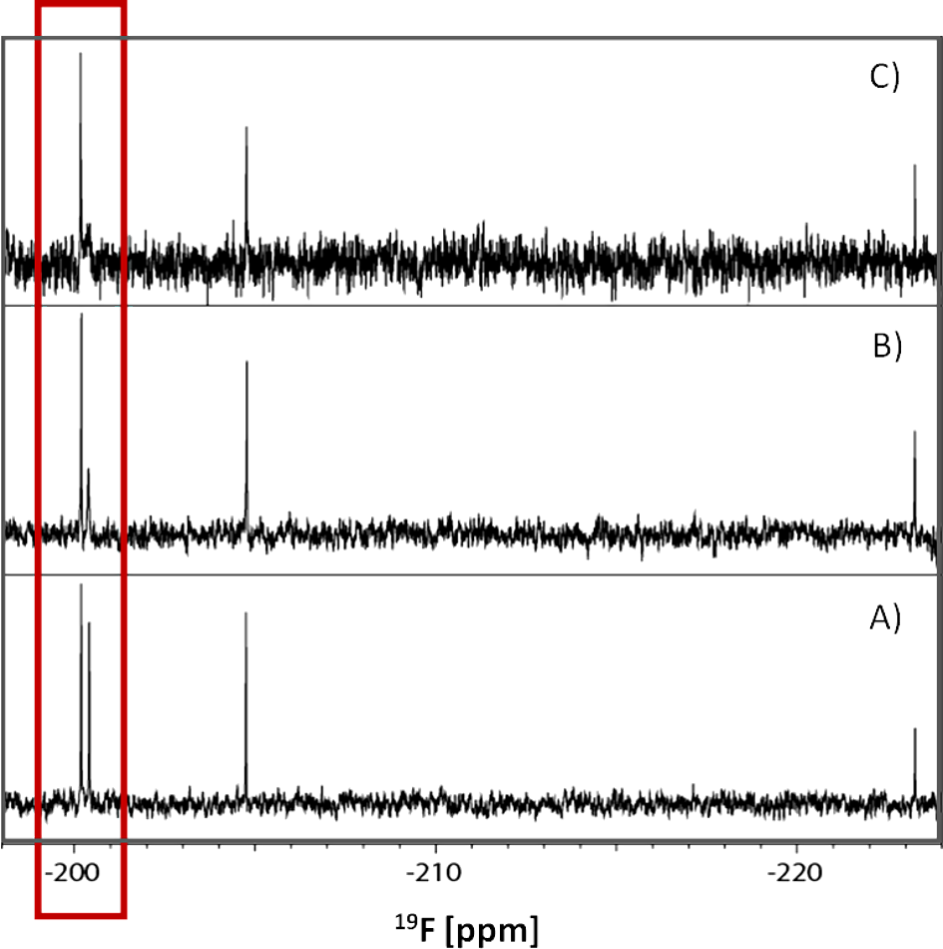
1-D ^19^F NMR: Experimental demonstration of differential binding of gluco- and manno-type 2-F-labeled maltose (2 mM) in the free form (A); bound to maltose-binding protein (200 µM) (B); and bound to MBP-V53 fusion protein (200 µM) (C). Highlighted area shows the gluco-type region. Spectra were recorded on a Bruker Avance DRX 600 MHz spectrometer by using a conventional 1-D pulse sequence. Up to 512 scans were acquired without signal suppression via T2 relaxation filter.

**Figure 4:**
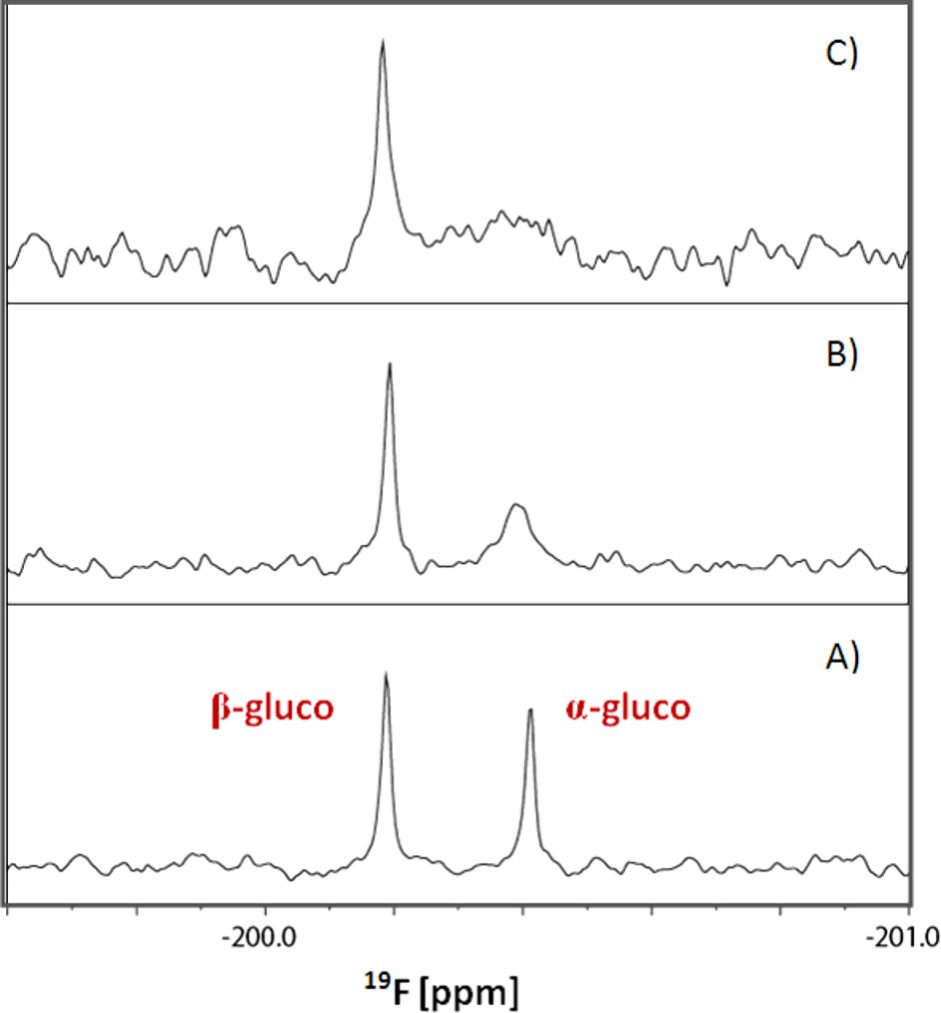
^19^F NMR expansion (of Figure 3) of the gluco-type region of the 2-F-maltose reporter system.

The binding properties of the two stereoisomers of 2-^19^F-labeled maltose (gluco- and manno-type) to the maltose-binding protein and a MBP-V53 fusion protein comprising five V3 modules of the LDL receptor in a linear tandem arrangement (V33333) were analyzed. As can be seen in Figure 3 and Figure 4, the stereoisomers of 2-F labeled maltose clearly exhibit different changes in the transverse relaxation rates upon addition of approx. 0.1 equiv of MBP. The significant change in line width was only observed for the interacting α-2-F-maltose. In contrast, the transverse relaxation remained nearly unchanged for the manno-type epimers and the β-gluco-type isomer. This observation corresponds to the anomeric preference described by Gehring et al. [40]. The numeric specifity of MBP with a 2.7-fold higher affinity for α- versus β-maltose was demonstrated by tritium NMR spectroscopy [40–42]. In addition, the β-anomer can be bound in two different modes, probably corresponding to the closed- and open-domain conformations of MBP; but only the α-anomer complex has been observed in X-ray structures of MBP with maltose [21].

Furthermore we used this technique for probing the interactions between 2-F-maltose and the MBP-V53 [43–44] fusion protein, which has almost twice the molecular weight of MBP alone. Upon the addition of 0.1 equiv again, the expected increase of the transverse relaxation rate was observed through the specific and significant binding of the α-gluco-type isomer to the MBP-V53 fusion protein. The larger resulting molecular weight is reflected in a further (proportional) increase of the line broadening (Figure 4). In a similar way, noncovalent protein–protein interactions would increase the effective molecular weight by transient binding and result in a consequently increased line width, which can be quantified to derive affinities. This clearly demonstrates both the binding selectivity of the α-gluco-type and the feasibility of the β-gluco-type and manno-type isomers, serving as internal reference compounds to rule out nonspecific binding and interactions (e.g., changes in viscosity). It should be noted that the detection limit of protein binding improves with decreasing ligand concentration, and thus even smaller protein and ligand concentrations can be used in the experiment [45]. Full exploitation of this effect, however, requires high performance ^19^F NMR probes (e.g., cryoprobes).

### Relative affinity studies using the 2-F-maltose reporter system

**Figure 5:**
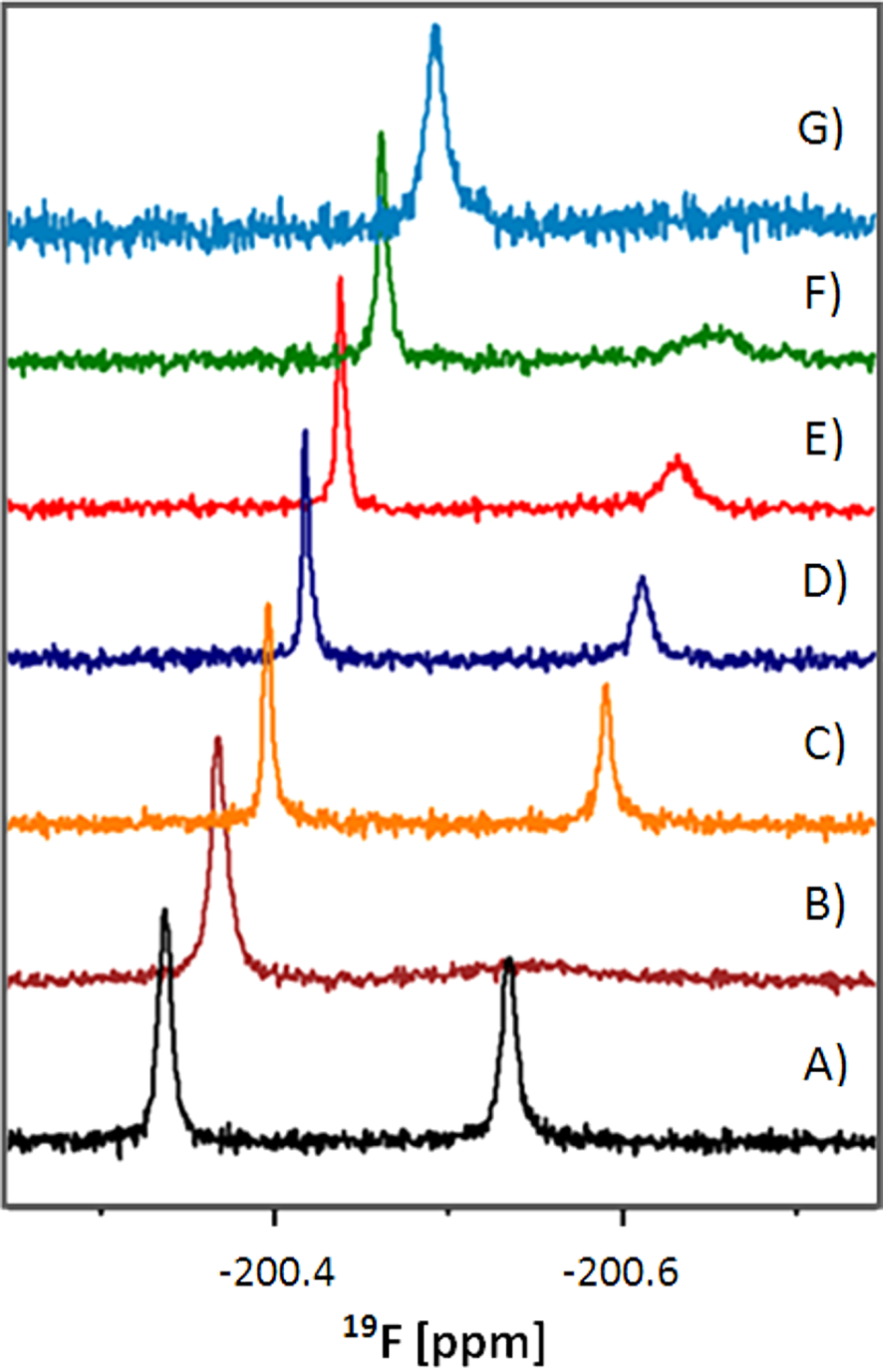
Competitive titration with the 2-F-maltose reporter system and ^19^F NMR: only the important section of the gluco-type isomers is shown. (A) 2-F-maltose, (B) 2-F-maltose bound to MBP, (C–G) addition of 0.125 equiv of the following maltose derivatives: (C) 6-F-maltose, (D) maltose, (E) α–maltosyl fluoride, (F) 6′-F-maltose, (G) 6′-F-“galacto”-maltose. Spectra were recorded on a Bruker Avance DRX 600 MHz spectrometer by using a conventional 1-D pulse sequence. Up to 128 scans were acquired without signal suppression via T2 relaxation filter.

The 2-F-maltose FAXS reporter system [5–7] was further used for studying the relative binding affinities of natural and artificial maltose derivatives to MBP. The initial experiments were performed with maltose, maltotriose, maltohexose and cellobiose, as well as the artificial α-methyl glucoside. The well-known ability of MBP to bind exclusively to linear maltooligosaccharides or maltodextrins of up to eight α(1→4)-linked glucose units was confirmed by competitive titration and ^19^F NMR experiments. The displacement of α-gluco-2-F-maltose was already observed by the addition of 0.04 equiv of maltose. Similar results were obtained for the malto-oligosaccharides, maltotriose and maltohexose as well. In contrast, α-methyl glucoside and cellobiose showed no binding. To specify the precise hydroxy groups that are directly involved in hydrogen bonding to MBP, further competition experiments were performed with different fluorinated maltose derivatives. Change, i.e., reduction in the line width of the α-2-F-maltose signal, could be observed if the competitor had a higher affinity than the α-2-F-maltose itself; caused by the release of α-2-F-maltose from the binding pocket of the maltose-binding protein. An overview of the results of the titration experiments is shown in Figure 5. The stepwise addition of equivalent amounts of single fluorinated maltose derivatives to the 2-F-maltose reporter system allows a direct comparison of the relative affinities of the competitors to MBP. The 6-F-maltose is the most efficient competitor with an affinity equal to maltose, α-maltosyl fluoride and 6′-F-maltose. The 6′-F-“galacto”-maltose derivative does not bind to MBP at all.

Note that the competitive binding experiments shown in Figure 5 allow for the direct extraction of dissociation constants, as was shown by Dalvit and co-workers [46–47]. This would offer additional valuable experimental possibilities for a quantitative analysis of protein–ligand interactions but is beyond the scope of the present paper. Fluorinated substrate analogues perturb the hydrogen bonding network in the substrate binding pocket to a certain extent. Therefore it is not always possible for the ligand to be bound with an optimal hydrogen-bonding geometry. These results are fully consistent with published X-ray data. For instance, in the case of 2-F-maltose, the 2-OH acts simultaneously as a hydrogen-bond acceptor for the Nε of Lys15 and as a bond donor to the carboxylate of Glu111, and the 2-F fluorine can only be a (limited) acceptor, thus leaving some of the H-bonds “frustrated”. It is worth comparing these findings with recently reported correlations between ^19^F chemical shifts and fluorine–protein interaction patterns [48–49]. Shielded fluorine atoms, due to their increased electron density, are preferentially involved in direct hydrogen-bonding interactions with donor groups of the protein. Although the 2-F fluorine is significantly shielded (about −200 ppm), and thus an ideal hydrogen-bond acceptor binding of 2-F-maltose is impaired due to the hydrogen-bond donor activity of the 2-OH group (to the carboxylate of Glu111). In that respect, introducing the fluorine into the 6-position results in a smaller energetic penalty (compared to the 2-F-maltose), because no direct H-bonds between the ligand and MBP are involved, and only indirect water-mediated interactions are concerned (data not shown). Therefore the affinity is higher in that case. Similar arguments apply in the other cases. It is, however, possible to “fine tune” the affinity between the ligand binding domain and the reporter ligand by using differently fluorinated maltose derivatives in which different hydroxy groups are substituted by fluorine. Thus the affinity of the reporter ligand can be “customized” for ligand competition assays or for specific studies of protein–ligand and protein–protein interactions to match the affinities between the interaction partners. For example, small affinities or proteins with a relatively low molecular weight are more easily detected with high-affinity ligands, whereas strongly interacting proteins or high-molecular-weight protein ligands can be better studied with low-affinity ligands.

## Conclusion

We have demonstrated that 2-deoxy-2-F-maltose can be effectively used as a reporter system to study protein-binding interactions by ^19^F NMR. The particular benefit of this novel reporter system is the simultaneous accessibility of reference molecules (nonbinding manno-type and β-gluco-type 2-F-maltose isomers), which serve as internal standards, to rule out nonspecific binding and interactions, and thus increasing the reliability of this method. The 2-F-maltose reporter system was used to study the ligand binding affinity to MBP. “Fine tuning” by the regioselective fluorination of single hydroxy groups of maltose was used to define the important hydroxy groups that are responsible for the hydrogen bonding network and therefore for binding to the protein. The results of the competitive titration are in perfect agreement with the X-ray data published [21] previously. Additionally, the different binding affinities of selectively ^19^F-labeled maltose derivatives to MBP illustrate how the recently established correlation between ^19^F chemical shift data and molecular interaction patterns [48–49] can be used to delineate details of protein–ligand interaction interfaces. Together with efficient synthetic approaches to fluorinated derivatives, this offers exciting perspectives for rational programs for drug design. Experiments to explore these possibilities are currently underway in our laboratories. Applications of the reporter system to biological material inherently giving strong background signals (e.g., membrane-bound protein receptors) should be straightforward, having the advantage that ^19^F signals can be detected with high sensitivity and without any background, and should broaden the applicability of this approach.

## Supporting Information

File 1 Detailed experimental procedures and spectral data of compounds **2**–**4**, **6**, **7**, **9**, **11**–**15**, **17**, **18**, **20**–**23**.
