# Supplementary material for: Synthesis of fluorinated maltose derivatives for monitoring protein interaction by 19F NMR
Source: Beilstein J Org Chem. 2012 Mar 27;8:448–55. doi: 10.3762/bjoc.8.51 (PMC3326624; doi:10.3762/bjoc.8.51)
Supplement: File 1 — Detailed experimental procedures and spectral data of compounds 2–4, 6, 7, 9, 11–15, 17, 18, 20–23. [file Beilstein_J_Org_Chem-08-448-s001.pdf]

Supporting Information

for

Synthesis of fluorinated maltose derivatives for monitoring protein interaction by ^{19}F NMR

Michaela Braitsch,[1] Hanspeter Kählig,[1] Georg Kontaxis,[2] Michael Fischer,[1] Toshinari Kawada,[3] Robert Konrat[2*] and Walther Schmid[1*]

Address: [1]Department of Organic Chemistry, University of Vienna, Währinger Strasse 38, A-1090 Vienna, Austria; [2]Department of Structural and Computational Biology, Max F. Perutz Laboratories, University of Vienna, Campus Vienna Biocenter 5, A-1030 Vienna, Austria and [3]Graduate School of Life and Environmental Sciences, Kyoto Prefectural University, Sakyo-Ku, Kyoto 606-8522, Japan

Email: Walther Schmid* - walther.schmid@univie.ac.at, Robert Konrat* - robert.konrat@univie.ac.at

*Corresponding author

Detailed experimental procedures and spectral data of compounds

2–4, 6, 7, 9, 11–15, 17, 18, 20–23

General methods

Solvents were purified by distillation and dried by standard procedures. Thin-layer chromatography (TLC) was performed on precoated silica gel plates 60 F254 (Merck), detected with UV light (254 nm) and ceric ammonium molybdate as well as 5% vanillin/sulfuric acid, and heated by a hotgun. For preparative column chromatography silica gel 60M (230-400 mesh, Macherey-Nagel) was used.

^{1}H and ^{13}C NMR spectra were recorded on Bruker AVANCE DPX 250, AV 400, DRX 400 WB or DRX 600 NMR spectrometers (Bruker BioSpin, Germany). Chemical shifts δ are expressed as parts per million (ppm) and were referenced to residual solvent signals at 7.26 ppm ($CDCl_3$) and 4.79 ppm (D_2O) for the proton NMR spectra as well as to the solvent signal 77.16 ppm ($CDCl_3$) or in D_2O to external dioxane at 67.2 ppm for ^{13}C NMR spectra. Coupling constants are quoted in Hz.

^{19}F NMR spectra were recorded on a Bruker AVANCE DRX 600 NMR spectrometer equipped with 5 mm QNP probe (^{1}H, ^{13}C, ^{19}F, ^{31}P) at a ^{19}F frequency of 564.69 MHz. Proton decoupling, when applied, was achieved by a Waltz-16 composite pulse decoupling sequence with a γB_1 of 1 kHz. ^{19}F resonances were referenced relative to external CCl_3F.

Mass spectra were recorded on electron spray ionization Finnigan MAT 8230 mass spectrometer.

Microwave heating was performed with a Biotage initiator synthesizer.

General procedures

1,2,3,6-Tetra-*O*-acetyl-4-*O*-(2',3',4',6'-tetra-*O*-acetyl-α-D-glucopyranosyl)-β-D-glucopyranoside (2):

To a cooled solution of maltose monohydrate **1** (20 g, 55.5 mmol) in dry pyridine (120 mL) containing *N,N*-dimethylaminopyridine (cat.), acetic anhydride (105 mL, 1.1 mol) was added dropwise. The mixture was stirred for 15 h at room temperature and was then poured into ice/water (1000 mL) to precipitate the peracetylated maltose. Filtration and drying under reduced pressure afforded the product as a colorless foam (36.5 g, 97%). ^{1}H NMR (400.13 MHz, $CDCl_3$) δ 5.73 (d, 1H, $J_{1,2}$ 8.2 Hz, H-1), 5.39 (d, 1H, $J_{1',2'}$ 4.0 Hz, H-1'), 5.34 (dd, 1H, $J_{2',3'}$ 10.5 Hz, $J_{3',4'}$ 9.7 Hz, H-3'), 5.28 (dd, 1H, $J_{2,3}$ 9.1 Hz, $J_{3,4}$ 8.7 Hz, H-3), 5.05 (dd, 1H, $J_{3',4'}$ 9.7 Hz, $J_{4',5'}$ 10.2 Hz, H-4'), 4.96 (dd, 1H, $J_{1,2}$ 8.2 Hz, $J_{2,3}$ 9.1 Hz, H-2), 4.85 (dd, 1H, $J_{1',2'}$ 4.0 Hz, $J_{2',3'}$ 10.5 Hz, H-2'), 4.44 (dd, 1H, $J_{5,6a}$ 2.5 Hz, $J_{6a,6b}$ 12.3 Hz, H-6a), 4.22 (dd, 1H, $J_{5,6b}$ 4.4, $J_{6a,6b}$ 12.3, H-6b), 4.23 (dd, 1H, $J_{5',6'a}$ 3.7 Hz, $J_{6'a,6'b}$ 12.4 Hz, H-6'a), 4.02 (dd, 1H $J_{3,4}$ 8.7 Hz, $J_{4,5}$ 9.6 Hz, H-4), 4.03 (dd, 1H, $J_{5'6'b}$ 2.4 Hz, $J_{6'a,6'b}$ 12.4 HzH-6'b), 3.93 (ddd, 1H, $J_{4'5'}$ 10.2 Hz, $J_{5'6'a}$ 2.4 Hz, $J_{5'6'b}$ 3.7 Hz, H-5'), 3.83 (ddd, $J_{5,6a}$ 2.6 Hz, $J_{5,6b}$ 4.4 Hz, $J_{4,5}$ 9.6 Hz, H-5), 2.13 (s, 3H, CH_3), 2.09 (s, 6H, 2 CH_3), 2.04 (s, 3H, CH_3), 2.02 (s, 3H, CH_3), 2.01 (s, 3H, CH_3), 2.00 (s, 3H, CH_3), 1.99 (s, 3H, CH_3); ^{13}C NMR (100.61 MHz, $CDCl_3$): δ 170.70 (C=O), 170.63 (C=O), 170.56 (C=O), 170.19 (C=O), 170.01 (C=O), 169.72 (C=O), 169.57 (C=O), 168.93 (C=O), 95.88 (C-1'), 91.43 (C-1), 75.39 (C-3), 73.16 (C-5), 72.61 (C-4), 71.10 (C-2), 70.16 (C-2'), 69.46 (C-3'), 68.74 (C-5'), 68.13 (C-4'), 62.68 (C-6), 61.61 (C-6'), 21.00 (CH_3), 20.93 (2 CH_3), 20.80 (CH_3), 20.71 (3 CH_3), 20.67 (CH_3). MS: Calcd for [$C_{28}H_{38}O_{19}$]: *m/z* 678:59: ESIMS found: $[M + Na]^+$ 700.9.

2,3,6-Tri-*O*-acetyl-4-*O*-(2',3',4',6'-tetra-*O*-acetyl-α-D-glucopyranosyl)-α-D-glucopyranosyl bromide (3):

Octaacetylmaltose **2** (10.5 g, 15.4 mmol) was dissolved in dry methylene chloride (DCM, 50 mL) and glacial acetic acid (50 mL). The solution was cooled to 0 °C and hydrobromic acid in glacial acetic acid (16.3 mL, 92.6 mmol) was added dropwise. The reaction mixture was stirred for 3 h at this temperature and quenched with ice/water (300 mL) and DCM (100 mL). The water layer was extracted with DCM (3 x 50 mL). The combined organic extracts were washed with water (70 mL), dried over $MgSO_4$, the solids were filtrated off and the solution was concentrated under reduced pressure. The light-sensitive α-bromide was obtained as a colorless foam (10.7 g, 99%). ^{1}H NMR (250.13 MHz, $CDCl_3$): δ 6.46 (d, 1H, $J_{1,2}$ 4.0 Hz, H-1), 5.57 (dd, 1H, $J_{2,3}$ 9.8 Hz, $J_{3,4}$ 8.9, H-3), 5.37 (d, 1H, $J_{1',2'}$ 4.0 Hz, H-1'), 5.33 (dd, 1H, $J_{2',3'}$ 10.5 Hz, $J_{3',4'}$ 9.4 Hz, H-3'), 5.03 (dd, 1H, $J_{3',4'}$ 9.4 Hz, $J_{4',5'}$ 10.1 Hz, H-4'), 4.82 (dd, 1H, $J_{1',2'}$ 4.0 Hz, $J_{2',3'}$ 10.5 Hz, H-2'), 4.67 (dd, 1H, $J_{1,2}$ 4.0 Hz, $J_{2,3}$ 9.9 Hz, H-2), 4.47 (dd, 1H, $J_{5,6a}$ 3.6 Hz, $J_{6a,6b}$ 13.6 Hz, H-6a), 4.25–4.17 (m, 3H, H-5, H-6b, H-6'a), 4.02 (dd, 1H, $J_{3,4}$ 8.9 Hz, $J_{4,5}$ 8.9 Hz, H-4), 4.01 (dd, 1H, $J_{5',6'b}$ 2.1 Hz, $J_{6'a,6'b}$ 12.9 Hz, H-6'b), 3.91 (ddd, 1H, $J_{4',5'}$ 10.1 Hz, $J_{5,6'a}$ 3.7 Hz, $J_{5',6'b}$ 2.1 Hz H-5'), 2.10 (s, 3H, CH_3), 2.05 (s, 3H, CH_3), 2.04 (s, 3H, CH_3), 2.03 (s, 3H, CH_3), 2.00 (s, 3H, CH_3), 1.98 (s, 3H, CH_3), 1.96 (s, 3H, CH_3). ^{13}C NMR (100.61 MHz, $CDCl_3$): δ 170.81 (C=O), 170.62 (C=O), 170.41 (C=O), 169.99 (2 C=O), 169.64 (C=O), 169.56 (C=O), 95.93 (C-1'), 86.19 (C-1), 72.69 (C-5), 72.49 (C-3), 71.75 (C-4), 71.17 (C-2), 70.16 (C-2'), 69.40 (C-3'), 68.80 (C-5'), 68.08 (C-4'), 62.00 (C-6), 61.50 (C-6'), 20.99 (CH_3), 20.90 (CH_3), 20.80 (CH_3), 20.76 (CH_3), 20.72 (3 CH_3).

3,6-Di-*O*-acetyl-4-*O*-(2',3',4',6'-tetra-*O*-acetyl-α-D-glucopyranosyl)-D-glucal (4):

To the vigorously stirred solution of α-maltosyl bromide **3** (2 g, 2.9 mmol) in ethyl acetate (10 mL), zinc powder (1.87 g, 28.6 mmol) and *N*-methyl imidazol (0.34 mL, 4.3 mmol) was added. The reaction mixture was heated under reflux for 4 h, cooled to room temperature and filtered over Celite to remove solid zinc. The organic layer was washed twice with saturated aqueous $NaHSO_4$ (20 mL), 10% aqueous $NaHCO_3$ (25 mL) and brine (25 mL), dried over $MgSO_4$ and concentrated under vacuum. The residue was purified by chromatography over silica gel ($CHCl_3$/Et_2O 1:2) to obtain the peracetylated maltal as a colorless foam (1.18 g, 74%). ^{1}H NMR (400.13 MHz, $CDCl_3$): δ 6.43 (dd, 1H, $J_{1,2}$ 6.1 Hz, $J_{1,3}$ 1.1 Hz, H-1), 5.50 (d, 1H, $J_{1',2'}$ 4.0 Hz, H-1'), 5.41 (dd, 1H, $J_{2',3'}$ 10.4 Hz, $J_{3',4'}$ 9.4 Hz, H-3'), 5.18 (m, 1H, H-3), 5.06 (dd, 1H, $J_{3',4'}$ 9.4 Hz, $J_{4',5'}$ 10.3 Hz, H-4'), 4.83 (dd, 1H, $J_{1',2'}$ 4.0 Hz, $J_{2',3'}$ 10.4 Hz, H-2'), 4.82 (dd, 1H, $J_{1,2}$ 6.1 Hz, $J_{2,3}$ 3.5 Hz,, H-2), 4.81–4.78 (m, 2H, H-2', H-2), 4.41–4.32 (m, 2H, H-6a, H-6b), 4.31–4.27 (m, 1H, H-5), 4.28 (dd, 1H, $J_{5',6'a}$ 4.2 Hz, $J_{6'a,6'b}$ 12.4 Hz, H-6'a), 4.10 (dd, 1H, $J_{5',6'b}$ 2.3, $J_{6'a,6'b}$ 12.4. H-6'b), 4.06–4.01 (m, 2H, H-4, H-5'), 2.12 (s, 3H, CH_3), 2.10 (s, 3H, CH_3), 2.054 (s, 3H, CH_3), 2.048 (s, 3H, CH_3), 2.03 (s, 3H, CH_3), 2.01 (6 s, 18H, CH_3); ^{13}C NMR (100.61 MHz, $CDCl_3$): δ 170.72 (C=O), 170.61 (C=O), 170.52 (C=O), 170.50 (C=O), 170.16 (C=O), 169.69 (C=O), 145.74 (C-1), 98.78 (C-2), 96.00 (C-1'), 74.26 (C-5), 72.70 (C-4), 70.59 (C-2'), 69.78 (C-3'), 69.66 (C-3), 68.45 (C-5'), 68.37 (C-4), 62.03 (C-6), 61.81 (C-6'), 21.24 (CH_3), 20.94 (CH_3), 20.82 (CH_3), 20.81 (CH_3), 20.75 (CH_3), 20.69 (CH_3). MS: Calcd for [$C_{24}H_{32}O_{15}$]: *m/z* 560.50: ESIMS found: $[M + Na]^+$ 582.9.

4-*O*-(α-D-glucopyranosyl)-2-deoxy-2-fluoro-D-gluco-and D-mannopyranoses (6):

To a solution of protected maltal **4** (131 mg, 0.23 mmol) in nitromethane (2.5 mL), Selectfluor™ (99 mg, 0.28 mmol) was added. The reaction mixture was stirred vigorously at room temperature for 15 h and then heated under reflux for 1 h. After cooling to room temperature, the solvent was removed under reduced pressure and the residue was redissolved in DCM (20 mL). The organic layer was washed with 10% aqueous $NaHCO_3$ solution (20 mL) and water (20 mL), dried over $MgSO_4$ and the solvent was removed under vacuum. The remaining material was purified by chromatography over silica gel (PE/EE 1:1). The mixture of product diastereomers **5** was isolated as a colorless foam (78 mg, 56%) with a gluco to manno ratio of 2:1. ^{19}F NMR (564.69 MHz, $CDCl_3$): glucose type, α-anomer δ -201.22 (dd, $J_{F,2}$ 49.7 Hz, $J_{F,3}$ 11.0 Hz), glucose type, β-anomer δ -200.41 (ddd, $J_{F,1}$ 2.4 Hz, $J_{F,2}$ 50.7 Hz, $J_{F,3}$ 12.9 Hz), mannose type, α-anomer δ -205.44 (dddd, $J_{F,1}$ 6.8 Hz, $J_{F,2}$ 50.0 Hz, $J_{F,3}$ 28.3 Hz, $J_{F,4}$ 2.3 Hz), mannose type, β-anomer δ -223.89 (ddd, $J_{F,1}$ 18.0 Hz, $J_{F,2}$ 50.9 Hz, $J_{F,3}$ 28.4 Hz).

Deprotection was performed according to the Zemplén protocol: The peracetylated 2-F-maltose mixture **5** (78 mg, 0.13 mmol) was suspended in dry methanol (2 mL). The sodium methoxide (0.5 mL of a freshly prepared 0.1 M stock solution) was added and stirred for 6 h at room temperature. By addition of dry ice or Dowex H^+ (pH 6–7), the excess of $NaOCH_3$ was quenched. Lyophilisation of this alcoholic solution yielded **6** as a colorless foam (45 mg, quant.). ^{19}F NMR (564.69 MHz, D_2O): glucose type, α-anomer δ -200.70 (dd, $J_{F,2}$ 49.3 Hz, $J_{F,3}$ 13.6 Hz), glucose type, β-anomer δ -200.48 (m), mannose type, α-anomer δ -205.08 (ddd, $J_{F,1}$ 7.5 Hz, $J_{F,2}$ 49.1 Hz, $J_{F,3}$ 31.4 Hz), mannose type, β-anomer δ -223.49 (ddd, $J_{F,1}$ 20.2 Hz, $J_{F,2}$ 51.4 Hz, $J_{F,3}$ 31.2 Hz).

2,3,6-Tri-*O*-acetyl-4-*O*-(2',3',4',6'-tetra-*O*-acetyl-α-D-glucopyranosyl)-β-D-glucopyranoside (7):

A solution of octaacetylmaltose **2** (5 g, 7.37 mmol) in dry DMF (25 mL) was stirred in the presence of hydrazine acetate (8.11 mmol) for 1½ h. (Hydrazine acetate was freshly prepared by combining equimolar amounts of hydrazine hydrate and acetic acid in methanol). The solvent was removed under reduced pressure to yield the anomeric mixture (α/β = 2:1) of the 1-hydroxy-maltose derivative **7** (4.4 g, 94%) as a colorless solid. **α-anomer:** ^{1}H NMR (600.13 MHz, $CDCl_3$): δ 5.58 (dd, 1H, $J_{3,2}$ 10.1 Hz, $J_{3,4}$ 9.0 Hz, H-3), 5.44 (d, 1H, $J_{1',2'}$ 4.1 Hz, H-1'), 5.37 (dd, 1H, $J_{3',2'}$ 10.6 Hz, $J_{3',4'}$ 9.5 Hz, H-3'), 5.36 (d, 1H, $J_{1,2}$ 3.6 Hz, H-1), 5.07 (dd, 1H, $J_{4',3'}$ 9.5 Hz, $J_{4',5'}$ 10.1 Hz, H-4'), 4.86 (dd, 1H, $J_{2,1}$ 4.1 Hz, $J_{2,3}$ 10.6 Hz, H-2'), 4.78 (dd, 1H, $J_{2,1}$ 3.6 Hz, $J_{2,3}$ 10.1 Hz, H-2), 4.50 (dd, 1H, $J_{5,6a}$ 2.21 Hz, $J_{6a,6b}$ 11.9 Hz, H-6a), 4.27–4.21 (m, 3H, H-6'a, H-6b, H-5), 4.05 (dd, 1H, $J_{5',6'b}$ 2.3 Hz, $J_{6'a,6'b}$ 12.4 Hz, H-6b), 3.99 (dd, 1H, $J_{3,4}$ 9.0 Hz, $J_{4,5}$ 9.5 Hz, H-4), 3.97 (m, 1H, H-5'), 2.15–2.00 (7 s, 21H, CH_3). ^{13}C NMR (150.90 MHz, $CDCl_3$): δ 170.97–169.58 (C=O), 95.68 (C-1'), 90.20 (C-1), 72.74 (C-4), 72.41 (C-3), 71.64 (C-2), 70.15 (C-2'), 69.54 (C-3'), 68.59 (C-5'), 68.15 (C-4'), 67.95 (C-5), 62.89 (C-6), 61.56 (C-6'), 21.19–20.73 (CH_3). **β-anomer:** ^{1}H NMR (600.13 MHz, $CDCl_3$): δ 5.40 (d, 1H, $J_{1',2}$ 4.1 Hz, H-1'), 5.35 (dd, 1H, $J_{3',2'}$ 10.5 Hz, $J_{3',4'}$ 9.5, H-3'), 5.30 (dd, 1H, $J_{3,2}$ 9.4 Hz, $J_{3,4}$ 8.9 Hz, H-3), 5.05 (dd, 1H, $J_{4',3'}$ 9.5 Hz, $J_{4',5'}$ 10.5 Hz, H-4'), 4.86 (dd, 1H, $J_{1',2'}$ 4.2 Hz, $J_{2',3'}$ 10.5 Hz, H-2'), 4.79 (d, 1H, $J_{1,2}$ 7.9 Hz, H-1), 4.73 (dd, 1H, $J_{2,1}$ 7.9 Hz, $J_{2,3}$ 9.4 Hz, H-2), 4.48 (dd, 1H, $J_{5,6a}$ 2.6 Hz, $J_{6a,6b}$ 12.1 Hz, H-6a), 4.27–4.21 (m, 2H, H-6b, H-6'a), 4.05 (dd, 1H, $J_{5',6'b}$ 2.4 Hz, $J_{6'a,6'b}$ 12.4 Hz, H-6'b), 4.00 (dd, 1H, $J_{3,4}$ 8.9 Hz, $J_{4,5}$ 9.6 Hz, H-4), 3.97–3.94 (m, 1H, H-5'), 3.74 (ddd, 1H, $J_{5,6a}$ 2.6, $J_{5,6b}$ 4.4, $J_{4,5}$ 9.6, H-5), 2.15–2.00 (7 s, 21H, CH_3). ^{13}C NMR (150.91 MHz, $CDCl_3$): δ 170.97–169.58

(C=O), 95.72 (C-1'), 95.12 (C-1), 74.85 (C-3), 74.00 (C-2), 72.80 (C-4), 72.59 (C-5), 70.13 (C-2'), 69.44 (C-3'), 68.71 (C-5'), 68.13 (C-4'), 62.99 (C-6), 61.61 (C-6'), 21.19–20.73 (CH_3). MS: Calcd for [$C_{26}H_{36}O_{18}$]: *m/z* 636.55: ESIMS found: $[M + Na]^+$ 658.9.

4-*O*-(α-D-glucopyranosyl)-α/β-D-glucopyranosyl fluorides (9):

Fluorination of the anomeric hydroxyl group was performed with DAST under an argon atmosphere. To a solution of peracetylated 1-hydroxy-maltose derivative **7** (1 g, 1.57 mmol) in dry DCM (25 mL), DAST (0.23 mL, 1.73 mmol) was added dropwise. The reaction mixture was stirred for 2 h at room temperature, quenched with 10% aqueous $NaHCO_3$ solution (15 mL), extracted with DCM (3x 15 mL), dried over $MgSO_4$ and concentrated under vacuum. The crude product was purified by chromatography over silica gel ($CHCl_3/Et_2O$ 1:1) to furnish the anomeric mixture of maltosyl fluoride **8** (0.89 g, 89%) as a colorless foam (α/β 1:3). Separation of the diastereomers could be achieved by HPLC. **α-anomer:** ^{19}F (564.69 MHz, $CDCl_3$): δ -149.21 ($J_{F,1}$ 53.1 Hz, $J_{F,2}$ 24.1 Hz). ^{1}H NMR (600.13 MHz, $CDCl_3$): δ 5.64 (dd, 1H, $J_{1,F}$ 53.1 Hz, $J_{1,2}$ 2.7 Hz, H-1), 5.53 (dd, 1H, $J_{2,3}$ 10.1 Hz, $J_{3,4}$ 9.0 Hz, H-3), 5.42 (d, 1H, $J_{1',2'}$ 4.0 Hz, H-1'), 5.34 (dd, 1H, $J_{3',2'}$ 10.6 Hz, $J_{3',4'}$ 9.5 Hz, H-3'), 5.05 (dd, 1H, $J_{4',3'}$ 9.5 Hz, $J_{4',5'}$ 10.2 Hz, H-4'), 4.85 (dd, 1H, $J_{2',1'}$ 4.0 Hz, $J_{2',3'}$ 10.6 Hz, H-2'), 4.82 (ddd, 1H, $J_{2,F}$ 24.1 Hz, $J_{2,1}$ 2.7 Hz, $J_{2,3}$ 10.1 Hz, H-2), 4.52 (dd, 1H, *J* 2.4 Hz, *J* 12.5 Hz, H-6a), 4.23 (dd, 2H, *J* 3.6 Hz, *J* 12.6 Hz), H-6a', H-6b), 4.16 (ddd, 1H, $J_{4,5}$ 10.0 Hz, $J_{5,6a}$ 2.4 Hz, $J_{5,6b}$ 3.6 Hz, H-5), 4.05 (dd, 1H, $J_{3,4}$ 9.0 Hz, $J_{4,5}$ 10.0 Hz, H-4), 4.03 (dd, 1H, $J_{5'6'b}$ 2.4 Hz, $J_{6'a,6'b}$ 12.5 Hz, H-6'b), 3.92 (ddd, $J_{4',5'}$ 10.2 Hz, $J_{5',6'a}$ 3.5 Hz, $J_{5',6'b}$ 2.4 Hz, H-5'), 2.14 (s, 3H, CH_3), 2.08 (s, 3H, CH_3), 2.06 (s, 3H, CH_3), 2.05(s, 3H, CH_3), 2.014 (s, 3H, CH_3), 2.01 (s, 3H, CH_3), 1.99 (s, 3H, CH_3). ^{13}C NMR (150.90 MHz, $CDCl_3$): δ 170.75 (C=O), 170.62 (C=O), 170.46 (C=O), 170.21 (C=O), 169.97 (C=O), 169.86 (C=O),

169.55 (C=O), 103.72 (d, $J_{1,F}$ 229.5 Hz, C-1), 95.79 (C-1'), 71.84 (C-3), 71.73 (C-4), 70.71 (d, $J_{2,F}$ 24.5 Hz, C-2), 70.32 (d, $J_{5,F}$ 3.9 Hz, C-5), 70.12 (C-2'), 69.36 (C-3'), 68.71 (C-5'), 68.00 (C-4'), 62.14 (C-6), 61.45 (C-6'), 21.15–20.61 (CH_3). **β-anomer:** ^{19}F (564.69 MHz, $CDCl_3$): δ -132.35 ($J_{F,1}$ 52.5 Hz, $J_{F,2}$ 8.7 Hz). ^{1}H NMR (600.13 MHz, $CDCl_3$): δ 5.42 (dd, 1H, $J_{1,2}$ 4.9, $J_{1,F}$ 52.5 Hz, H-1), 5.41 (d, 1H, $J_{1',2'}$ 4.0 Hz, H-1'), 5.37 (dd, 1H, $J_{2',3'}$ 10.6 Hz, $J_{3',4'}$ 9.5 Hz, H-3'), 5.13 (dd, 1H, $J_{2,3}$ 6.4 Hz, $J_{3,4}$ 7.8 Hz, H-3), 5.06 (dd, 1H, $J_{3',4'}$ 9.5 Hz, $J_{4',5'}$ 10.3 Hz, H-4'), 4.95 (dd, 1H, $J_{2,F}$ 8.7 Hz, $J_{2,1}$ 4.9 Hz, $J_{2,3}$ 6.3 Hz, H-2), 4.84 (dd, 1H, $J_{2',1'}$ 4.0 Hz, $J_{2',3'}$ 10.6 Hz, H-2'), 4.54 (dd, 1H, $J_{5,6a}$ 3.4 Hz, $J_{6a,6b}$ 12.2 Hz, H-6a), 4.24 (dd, 1H, $J_{5,6'a}$ 4.1 Hz, $J_{6'a,6'b}$ 12.5 Hz, H-6'a), 4.22 (dd, 1H, $J_{5,6b}$ 4.6 Hz, $J_{6a,6b}$ 12.2 Hz, H-6b), 4.15 (ddd, 1H, $J_{4,3}$ 7.8 Hz, $J_{4,5}$ 8.7 Hz, $J_{4,F}$ 0.6 Hz, H-4), 4.07 (dd, 1H, $J_{5,6'b}$ 2.4 Hz, $J_{6'a,6'b}$ 12.5 Hz, H-6'b), 4.00–3.98 (m, 2H, H-5, H-5'), 2.15 (s, 3H, CH_3), 2.11 (s, 3H, CH_3), 2.10 (s, 3H, CH_3), 2.05 (s, 3H, CH_3), 2.04 (s, 3H, CH_3), 2.03 (s, 3H, CH_3), 2.00 (s, 3H, CH_3). ^{13}C NMR (150.91 MHz, $CDCl_3$): δ 170.68 (C=O), 170.58 (C=O), 170.56 (C=O), 170.16 (C=O), 170.11 (C=O), 169.57 (C=O), 169.49 (C=O), 105.61 ($J_{1,F}$ 219.8 Hz, C-1), 96.06 (C-1'), 74.17 ($J_{3,F}$ 5.5 Hz, C-3), 72.45 ($J_{5,F}$ 1.7 Hz, C-5), 72.14 (C-4), 71.37 ($J_{2,F}$ 31.9 Hz, C-2), 70.30 (C-2'), 69.45 (C-3'), 68.73 (C-5'), 68.15 (C-4'), 62.79 (C-6), 61.65 (C-6'), 20.98–20.68 (CH_3). MS: Calcd for [$C_{26}H_{35}FO_{17}$]: *m/z* 638.54: ESIMS found: $[M + Na]^+$ 661.0.

According to the procedure described for compound **6**, the deprotection of peracetylated α-maltosyl fluoride (100 mg, 0.16 mmol) with sodium methoxide (0.4 equiv) afforded α-maltosyl fluoride **9** in quantitative yield.

α-anomer: ^{19}F (564.69 MHz, D_2O): δ -150.92($J_{F,1}$ 53.5 Hz, $J_{F,2}$ 26.4 Hz). ^{1}H NMR (600.13 MHz, D_2O): δ 5.69 (dd, 1H, $J_{1,F}$ 53.5 Hz, $J_{1,2}$ 2.8 Hz, H-1), 5.42 (d, 1H, $J_{1',2'}$ 3.9 Hz, H-1'), 4.00 (dd, 1H, $J_{3,2}$ 9.9 Hz, $J_{3,4}$ 9.2 Hz, H-3), 3.95 (ddd, 1H, $J_{4,5}$ 10.0 Hz, $J_{5,6a}$ 2.0 Hz, $J_{5,6b}$ 4.2 Hz, H-5), 3.88 (dd, 1H, $J_{5,6a}$ 2.0 Hz, $J_{6a,6b}$ 12.4 Hz, H-6a), 3.85 (dd, 1H, $J_{5',6'a}$ 2.0 Hz, $J_{6'a,6'b}$ 12.4 Hz, H-

6'a), 3.83 (dd, 1H, $J_{5,6b}$ 4.2 Hz, $J_{6a,6b}$ 12.4 Hz, H-6b), 3.76 (dd, 1H, $J_{5',6'b}$ 5.1 Hz, $J_{6'a,6'b}$ 12.4 Hz, H-6'b), 3.75 (dd, 1H, $J_{3,4}$ 9.2 Hz, $J_{4,5}$ 10.0 Hz, H-4), 3.71 (ddd, 1H, $J_{4',5'}$ 9.9 Hz, $J_{5',6'a}$ 2.0 Hz, $J_{5'6'b}$ 5.1 Hz, H-5'), 3.68 (dd, 1H, $J_{3',2'}$ 9.9 Hz, $J_{3',4'}$ 9.9 Hz, H-3'), 3.65 (ddd, 1H, $J_{2,F}$ 26.4 Hz, $J_{2,1}$ 2.8 Hz, $J_{2,3}$ 9.9 Hz H-2), 3.58 (dd, 1H, $J_{2',1'}$ 3.9 Hz, $J_{2',3'}$ 9.9 Hz, H-2'), 3.41 (dd, 1H, $J_{4',3'}$ 9.9 Hz, ,$J_{4',5'}$ 9.9 Hz, H-4'). ^{13}C NMR (150.90 MHz, D_2O): δ 107.47 (d, $J_{1,F}$ 223.3 Hz, C-1), 100.09 (C-1'), 75,97 (C-4), 73.23(C-3), 73.17 (C-5'), 73.07 (C-3'), 73.04 (d, $J_{5,F}$ 3.2 Hz, C-5), 72.06 (C-2'), 71.23 (d, $J_{2,F}$ 24.9 Hz, C-2), 69.63 (C-4'), 60.78 (C-6'), 60.45 (C-6).

1,2,3,6-Tetra-*O*-acetyl-4-*O*-(2',3'-di-*O*-acetyl-4',6'-benzylidene-α-D-glucopyranosyl)-D-glucopyranoside (11):

In a 100 mL round-bottomed flask maltose monohydrate **1** (5 g, 13.9 mmol), α,α-dimethoxytoluene (4.9 mL) and p-toluene sulfonic acid (0.25 g) were suspended in dry DMF (45 mL). The flask was attached to a rotary evaporator. The reaction mixture was stirred by rotation for 5 hours at 50 °C and under 39 mbar vacuum. After neutralization with conc. NH_3 (pH~7) the solvents were removed under reduced pressure. The crude product was purified by chromatography over silica ($CHCl_3$/MeOH 3:1) to yield benzylidenemaltose **10** in 79% (4.7 g). According to the procedure described for compound **2**, the peracetylation of benzylidenemaltose (4.3 g, 10 mmol) with acetic anhydride (19 mL, 200 mmol) and catalytic amount of DMAP in dry pyridine (20 mL), followed by purification by chromatography over silica gel (PE/EE 1:1) afforded compound **11** in 93% yield (6.4 g). **α-anomer:** ^{1}H NMR (400.13 MHz, $CDCl_3$): δ 7.44–7.41 (m, 2H, H-2,6 phenyl), 7.35–7.33 (m, 3H, H-3,4,5 phenyl), 6.23 (d, 1H, $J_{1,2}$ 3.7 Hz, H-1), 5.52 (dd, 1H, $J_{3,4}$ 8.7 Hz, $J_{3,2}$ 10.1 Hz, H-3), 5.47 (dd, 1H, $J_{2',3'}$ 10.2 Hz, $J_{3',4'}$ 9.6 Hz, H-3'), 5.47 (s, 1H, CH-phenyl), 5.37 (d, 1H, $J_{1',2'}$ 4.2 Hz, H-1'), 4.97 (dd, 1H, $J_{2,1}$ 3.7 Hz, $J_{2,3}$ 10.1 Hz, H-2), 4.90 (dd, 1H, $J_{2',1'}$ 4.2 Hz, $J_{2',3'}$ 10.2 Hz, H-2'), 4.50 (dd, 1H, $J_{5,6a}$ 2.4 Hz, $J_{6a,6b}$ 12.4 Hz, H-6a),

4.28–4.22 (m, 2H, H-6b, H-6'a), 4.12 (ddd, 1H, $J_{4,5}$ 9.7 Hz, $J_{5,6a}$ 2.4 Hz, $J_{5,6b}$ 3.5 Hz, H-5), 4.04 (dd, 1H, $J_{3,4}$ 8.7 Hz, $J_{4,5}$ 9.7 Hz, H-4), 3.89–3.81 (m, 1H, H-5'), 3.71 (dd, 1H, $J_{6'b,5'}$ 4.3 Hz, $J_{6'b,6'a}$ 10.2 Hz, H-6'b), 3.64 (dd, 1H, $J_{4',3'}$ 9.6 Hz, $J_{4',5'}$ 9.6 Hz, H-4'), 2.22 (s, 3H, CH_3), 2.11 (s, 3H, CH_3), 2.09 (s, 3H, CH_3), 2.05 (s, 3H, CH_3), 2.03 (s, 3H, CH_3), 1.99 (s, 3H, CH_3). ^{13}C NMR (100.61 MHz, $CDCl_3$): δ 170.97 (C=O), 170.04 (C=O), 169.88 (C=O), 169.82 (C=O), 169.77 (C=O), 169.09 (C=O), 136.81 (C1 phenyl.), 129.26 (C-4 phenyl), 128.35 (C-3,5 phenyl), 126.34 (C-2,6 phenyl), 101.78 (CH-phenyl), 96.89 (C-1'), 89.02 (C-1), 78.89 (C-4'), 72.65 (C-4), 72.53 (C-3), 71.06 (C-2'), 70.36 (C-5), 69.91 (C-2), 68.58 (C-3'), 68.55 (C-6'), 64.00 (C-5'), 62.43 (C-6), 21.14–20.58 (CH_3). **β-anomer:** ^{1}H NMR (400.13 MHz, $CDCl_3$): δ 7.44–7.41 (m, 2H, H-2,6 phenyl), 7.35–7.33 (m, 3H, H-3,4,5 phenyl), 5.74 (d, 1H, $J_{1,2}$ 8.2 Hz, H-1), 5.47 (s, 1H, CH-phenyl), 5.45 (dd, 1H, $J_{2',3'}$ 10.2 Hz, $J_{3',4'}$ 9.6 Hz, H-3'), 5.35 (d, 1H, $J_{1',2'}$ 4.2, H-1'), 5.30 (dd, 1H, $J_{3,2}$ 9.2 Hz, $J_{3,4}$ 8.8 Hz, H-3), 4.97 (dd, 1H, $J_{2,1}$ 8.2 Hz, $J_{2,3}$ 9.2 Hz, H-2), 4.88 (dd, 1H, $H_{2',1'}$ 4.2 Hz, $H_{2',3'}$ 10.2 Hz, H-2'), 4.49 (dd, 1H, $J_{5,6}$ 2.6 Hz, $J_{6a,6b}$ 12.3 Hz, H-6a), 4.28–4.22 (m, 2H, H-6b, H-6'a), 4.04 (dd, 1H, $J_{3,4}$ 8.8 Hz, $J_{4,5}$ 9.9 Hz, H-4), 3.89–3.81 (m, 2H, H-5, H-5'), 3.73 (dd, 1H, $J_{6'b,5'}$ 4.3 Hz, $J_{6'b,6'a}$ 10.3 Hz, H-6'b), 3.62 (dd, 1H, $J_{4',3'}$ 9.6 Hz, $J_{4',5'}$ 9.6 Hz, H-4'), 2.10 (s, 3H, CH_3), 2.09 (s, 3H, CH_3), 2.06 (s, 3H, CH_3), 2.04 (s, 3H, CH_3), 2.02 (s, 3H, CH_3), 2.01 (s, 3H, CH_3). ^{13}C NMR (100.61 MHz, $CDCl_3$): δ 170.95 (C=O), 170.37 (C=O), 170.36 (C=O), 170.17 (C=O), 170.05 (C=O), 168.90 (C=O), 136.81 (C-1 phenyl), 129.26 (C-4 phenyl), 128.35 (C-3,5 phenyl), 126.34 (C-2,6 phenyl), 101.78 (CH-phenyl), 96.76 (C-1'), 91.40 (C-1), 78.91 (C-4'), 75.47 (C-3), 73.19 (C-5), 72.69 (C-4), 71.17 (C-2'), 70.98 (C-2), 68.58 (C-3'), 68.55 (C-6'), 63.91 (C-5'), 62.51 (C-6), 21.14–20.58 (CH_3). MS: Calcd for [$C_{31}H_{38}O_{17}$]: *m/z* 682.62: ESIMS found: $[M + Na]^+$ 705.7.

1,2,3,6-Tetra-*O*-acetyl-4-*O*-(2',3'-di-*O*-acetyl-4'-*O*-benzyl-α-D-glucopyranosyl)-D-glucopyranoside (12):

In an oven-dried round-bottomed reaction flask compound **11** (123 mg, 0.18 mmol) was dissolved in freshly distilled THF under argon atmosphere. The solution was cooled to -70 °C and was then treated with borane-THF complex (0.9 mL of a 1M solution in THF). After 15 min, the reaction mixture was treated with Bu_2BOTf (0.45 mL of a 1M solution in DCM) and stirred for 4 hours. The temperature was brought to 10 °C over this period of time. The reaction was quenched with NH_3 (0.2 mL) and dropwise addition of MeOH (6.0 mL) until effervescence ceased. The product was concentrated in vacuum and purified by flash column chromatography (silica gel, PE/EE 1:1) to yield the regioselectively deprotected compound **12** in 56% (69 mg). **α-anomer:** ^{1}H NMR (400.13 MHz, $CDCl_3$): δ 7.35–7.24 (m, 5H, H phenyl), 6.21 (d, 1H, $J_{1,2}$ 3.7 Hz, H-1), 5.48 (dd, 1H, $J_{3,4}$ 8.6 Hz, $J_{3,2}$ 10.1 Hz, H-3), 5.39 (dd, 1H, *J* 10.7 Hz, *J* 8.7 Hz, H-3'), 5.34 (d, 1H, $J_{1',2'}$ 4.0 Hz, H-1'), 4.94 (dd, 1H, $J_{2,1}$ 3.7 Hz, $J_{2,3}$ 10.0 Hz, H-2), 4.74 (dd, 1H, $J_{2',1'}$ 4.0 Hz, $J_{2',3'}$ 10.7 Hz, H-2'), 4.62 (d, 1H, *J* 11.4 Hz, CH_2-phenyl), 4.58 (d, 1H, *J* 11.4 Hz, CH_2-phenyl), 4.41 (dd, 1H, $J_{6a,5}$ 2.5 Hz, $J_{6a,6b}$ 12.3 Hz, H-6a), 4.16 (dd, 1H, $J_{6a,5}$ 3.3 Hz, $J_{6b,6a}$ 12.3 Hz, H-6b), 4.07 (ddd, 1H, $J_{4,5}$ 9.9 Hz, $J_{5,6a}$ 2.5 Hz, $J_{5,6b}$ Hz, H-5), 4.00 (dd, 1H, $J_{3,4}$ 8.6 Hz, $J_{4,5}$ 9.9 Hz, H-4), 3.77–3.71 (m, 2H, H-6'a, H-6'b), 3.70–3.61 (m, 2H, H-4', H-5'), 2.20–1.92 (CH_3). ^{13}C NMR (100.61 MHz, $CDCl_3$): δ 170.97 (C=O), 170.68 (C=O), 169.94 (C=O), 169.91 (C=O), 169.75 (C=O), 169.07 (C=O), 137.54 (C-1 phenyl), 128.64 / 128.15 (C-2,3,5,6 phenyl), 128.16 (C-4 phenyl), 96.16 (C-1'), 88.96 (C-1), 75.33 (C-4'), 74.85 (CH_2-phenyl), 72.54 (C-3), 72.43 (C-4), 72.30 (C-5'), 71.20 (C-3'), 70.83 (C-2'), 70.29 (C-5), 69.83 (C-2), 62.61 (C-6), 61.25 (C-6'), 21.11–20.51 (CH_3). **β-anomer:** ^{1}H NMR (400.13 MHz, $CDCl_3$): δ 7.35–7.24 (m, 5H, H

phenyl), 5.71 (d, 1H, $J_{1,2}$ 8.1 Hz, H-1), 5.41 (dd, 1H, $J_{3,2}$ 10.7 Hz, $J_{3,4}$ 8.7, H-3'), 5.31 (d, 1H, $J_{1',2'}$ 4.0, H-1'), 5.27 (dd, 1H, $J_{3,2}$ 9.3 Hz, $J_{3,4}$ 8.7 Hz, H-3), 4.94 (dd, 1H, $J_{2,1}$ 8.1 Hz, $J_{2,3}$ 9.3 Hz, H-2), 4.73 (dd, 1H, $H_{2',1'}$ 4.0 Hz, $H_{2',3'}$ 10.7 Hz, H-2'), 4.61 (d, 1H, *J* 11.4 Hz, CH_2-Phenyl), 4.47 (d, 1H, *J* 11.4 Hz, CH_2-Phenyl), 4.42 (dd, 1H, $J_{6a,5}$ 2.5 Hz, $J_{6a,6b}$ 12.4 Hz, H-6a), 4.16 (dd, 1H, $J_{6a,5}$ 4.3 Hz, $J_{6b,6a}$ 12.4 Hz, H-6b), 3.79 (ddd, 1H, $J_{4,5}$ 9.6 Hz, $J_{5,6a}$ 2.5 Hz, $J_{5,6b}$ 4.3 Hz, H-5), 3.77–3.71 (m, 2H, H-6'a, H-6'b), 3.70–3.61 (m, 2H, H-4', H-5'), 2.20–1.92 (CH_3). ^{13}C NMR (100.61 MHz, $CDCl_3$): δ 170.94 (C=O), 170.71 (C=O), 170.06 (C=O), 169.98 (C=O), 169.77 (C=O), 168.85 (C=O), 137.54 (C-1 phenyl), 128.63 / 128.14 (C-2,3,5,6 phenyl), 128.18 (C-4 phenyl), 96.13 (C-1'), 91.35 (C-1), 75.41 (C-3), 75.34 (C-4'), 74.81 (CH_2-phenyl), 73.13 (C-5), 72.46 (C-5'), 72.38 (C-4), 71.20 (C-3'), 71.09 (C-2), 70.79 (C-2'), 62.73 (C-6), 61.25 (C-6'), 21.11–20.51 (CH_3). MS: Calcd for [$C_{31}H_{40}O_{17}$]: *m/z* 684.64: ESIMS found: $[M + Na]^+$ 707.2.

1,2,3,6-Tetra-*O*-acetyl-4-*O*-(2',3'-di-*O*-acetyl-4'-*O*-benzyl-6'-deoxy-6'-fluoro-α-D-glucopyranosyl)-D-glucopyranoside (13):

A solution of compound **12** (62 mg, 0.09 mmol), DAST (0.024 mL, 0.18 mmol) and collidine (0.024 mL, 0.18 mmol) in anhydrous DCM was heated in the microwave generator for 60 minutes at 80 °C. TLC control showed no remaining starting material. The reaction was quenched with saturated $NaHCO_3$. The solution was extracted thrice with DCM (10 mL). The organic layers were washed with water, dried over $MgSO_4$ and the solvents were removed under reduced pressure. The crude product was purified by flash column chromatography (silicagel, PE/EE 1:1) to give 49 mg (79%) of compound **13**. **α-anomer:** ^{19}F (564.69 MHz, $CDCl_3$): δ -234.82 (td, $J_{F,6'}$ 47.6 Hz, $J_{F,5'}$ 28.8 Hz). ^{1}H NMR (600.13 MHz, $CDCl_3$): δ 7.34–7.22 (m, 5H, phenyl), 6.21 (d, 1H, $J_{1,2}$ 3.7 Hz, H-1), 5.48 (dd, 1H, $J_{3,4}$ 8.5 Hz, $J_{3,2}$ 10.1 Hz, H-3), 5.42 (dd, 1H,

$J_{3',2'}$ 10.6 Hz, $J_{3',4'}$ 9.3 Hz, H-3'), 5.38 (d, 1H, $J_{1',2'}$ 4.0 Hz, H-1'), 4.94 (dd, 1H, $J_{2,1}$ 3.7 Hz, $J_{2,3}$ 10.1 Hz, H-2), 4.78 (dd, 1H, $J_{2',1'}$ 4.0 Hz, $J_{2',3'}$ 10.6 Hz, H-2'), 4.61 (d, 1H, J 11.3 Hz, CH_2-phenyl), 4.59 (ddd, 1H, $J_{F,6'a}$ 47.6 Hz, $J_{6'a,5'}$ 2.7 Hz, $J_{6'a,6'b}$ 10.6 Hz, H-6'a), 4.58 (d, 1H, J 11.3 Hz, CH_2-phenyl), 4.49 (ddd, 1H, $J_{F,6'b}$ 47.6 Hz, $J_{6'a,5'}$ 1.4 Hz, $J_{6'b,6'a}$ 10.6 Hz, H-6'b), 4.36 (dd, 1H, $J_{6a,5}$ 2.1 Hz, $J_{6a,6b}$ 12.4 Hz, H-6a), 4.15 (dd, 1H, $J_{6b,5}$ 3.0 Hz, $J_{6b,6a}$ 12.4 Hz, H-6b), 4.06–4.01 (m, 2H, H-4, H-5), 3.81–3.69 (m, 1H, H-5'), 3.65 (dd, 1H, $J_{4',3'}$ 9.3 Hz, $J_{4',5'}$ 9.9 Hz , H-4'), 2.20 (s, 3H, CH_3), 2.08 (s, 3H, CH_3), 2.04 (s, 3H, CH_3), 1.99 (s, 3H, CH_3), 1.97 (s, 3H, CH_3), 1.93 (s, 3H, CH_3). ^{13}C NMR (100.61 MHz, $CDCl_3$): δ 170.85–168.85 (C=O), 137.27 (C-1 phenyl), 128.67 / 128.11 (C-2,3,5,6 phenyl) 128.25 (C-4 phenyl), 96.13 (C-1'), 88.97 (C-1), 81.38 (d, $J_{F,6'}$ 173.9 Hz, C-6'), 75.08 (CH_2-phenyl), 74.86 (d, $J_{F,4'}$ 6.1 Hz, C-4'), 72.51 (C-3), 72.15 (C-4), , 71.30 (C5'), 71.16 (C3'), 70.54 (C2'), 70.21 (C5), 69.85 (C2), 62.39 (C-6), 21.10–20.51 (CH_3).

β-anomer: ^{19}F (564.69 MHz, $CDCl_3$): δ -234.90 (td, $J_{F,6'}$ 47.7 Hz, $J_{F,5'}$ 28.9 Hz). ^{1}H NMR (600.13 MHz, $CDCl_3$): δ 7.34–7.22 (m, 5H, phenyl), 5.72 (d, 1H, $J_{1,2}$ 8.1 Hz, H-1), 5.39 (dd, 1H, $J_{3',2'}$ 10.5 Hz, $J_{3',4'}$ 9.3, H-3'), 5.34 (d, 1H, $J_{1',2'}$ 4.0, H-1'), 5.27 (dd, 1H, $J_{3,2}$ 9.1 Hz, $J_{3,4}$ 8.7 Hz, H-3), 4.94 (dd, 1H, $J_{2,1}$ 8.1 Hz, $J_{2,3}$ 9.1 Hz, H-2), 4.77 (dd, 1H, $H_{2',1'}$ 4.0 Hz, $H_{2',3'}$ 10.5 Hz, H-2'), 4.60 (d, 1H, J 11.3 Hz, CH_2-phenyl), 4.59 (ddd, 1H, $J_{F,6'a}$ 47.7 Hz, $J_{6'a,5'}$ 2.7 Hz, $J_{6'a,6'b}$ 10.6 Hz, H-6'a), 4.49 (ddd, 1H, $J_{F,6'b}$ 47.7 Hz, $J_{6'a,5'}$ 1.4 Hz, $J_{6'b,6'a}$ 10.6 Hz, H-6'b), 4.47 (d, 1H, J 11.3 Hz, CH_2-phenyl), 4.37 (dd, 1H, $J_{6a,5}$ 2.5 Hz, $J_{6a,6b}$ 12.4 Hz, H-6a), 4.155 (dd, 1H, $J_{6b,5}$ 4.2 Hz, $J_{6b,6a}$ 12.4 Hz, H-6b), 4.02 (dd, 1H, $J_{4,3}$ 8.7 Hz, $J_{4,5}$ 9.6 Hz, H-4), 3.79 (ddd, 1H, $J_{4,5}$ 9.6 Hz, $J_{5,6a}$ 2.5 Hz, $J_{5,6b}$ 4.2 Hz, H-5), 3.80–3.70 (m, 1H, H-5'), 3.64 (dd, 1H, $J_{4',3'}$ 9.3 Hz, $J_{4',5'}$ 9.9 Hz, H-4'), 2.075 (s, 3H, CH_3), 2.07 (s, 3H, CH_3), 2.02 (s, 3H, CH_3), 1.99 (s, 3H, CH_3), 1.98 (s, 3H, CH_3), 1.92 (s, 3H, CH_3). ^{13}C NMR (100.61 MHz, $CDCl_3$): δ 170.85–168.85 (C=O), 137.27 (C-1 phenyl), 128.66 / 128.09 (C-2,3,5,6 phenyl), 128.23 (C-4 phenyl), 96.12 (C-1'), 91.32 (C-1),

81.38 (d, $J_{F,6'}$ 173.9 Hz, C-6'), 75.50 (C-3), 75.04 (CH_2-phenyl), 74.85 (d, $J_{F,4'}$ 6.1 Hz, C-4'), 73.04 (C-5), 72.42 (C-4), 71.16 (C-3'), 71.10 (C-2), 70.51 (C-2'), 62.49 (C-6), 21.10-20.51 (CH_3). MS: Calcd for [$C_{31}H_{39}FO_{16}$]: *m/z* 686.63: ESIMS found: $[M + Na]^+$ 709.2.

1,2,3,6-Tetra-*O*-acetyl-4-*O*-(2',3'-di-*O*-acetyl-6'-deoxy-6'-fluoro-α-D-glucopyranosyl)-D-glucopyranoside (14):

To a solution of compound **13** (100 mg, 0.15 mmol) in 10 mL ethyl acetate, 10 mg of Pd/C (10 mg) was added. The reaction mixture was flushed twice with argon and stirred over the weekend under hydrogen atmosphere at room temperature. The suspension was filtered over a celite pad and the concentrated in vacuum. Purification by column chromatography (silica gel, PE/EE 1:2) afforded compound **14** in 64% yield (56 mg). **α-anomer:** ^{19}F (564.69 MHz, $CDCl_3$): δ -235.52 ($J_{F,6'a}$ 47.4 Hz, $J_{F,6'b}$ 47.4 Hz, $J_{F,5'}$ 25.4 Hz). ^{1}H NMR (600.13 MHz, $CDCl_3$): δ 6.21 (d, 1H, $J_{1,2}$ 3.7 Hz, H-1), 5.50–5.47 (m, 1H, H-3), 5.40 (d, 1H, $J_{1',2'}$ 3.9 Hz, H-1'), 5.21 (dd, 1H, $J_{3',2'}$ 10.6 Hz, $J_{3',4'}$ 9.3 Hz, , H-3'), 4.94 (dd, 1H, $J_{2,1}$ 3.7 Hz, $J_{2,3}$ 10.2 Hz, H-2), 4.77 (dd, 1H, $J_{2',1'}$ 3.9 Hz, $J_{2',3'}$ 10.6 Hz, H-2'), 4.62 (ddd, 1H, $J_{F,6'a}$ 47.4 Hz, $J_{6'a,5'}$ 3.9 Hz, $J_{6'a,6'b}$ 10.3 Hz, H-6'a), 4.54 (ddd, 1H, $J_{F,6'}$ 47.4 Hz, $J_{6'b,5'}$ 1.7 Hz, $J_{6'b,6'a}$ 10.3 Hz, H-6'b), 4.40 (dd, 1H, $J_{6a,5}$ 1.8 Hz, $J_{6a,6b}$ 12.4 Hz, H-6a), 4.17 (dd, 1H, $J_{6b,5}$ 2.9 Hz, $J_{6b,6a}$ 12.4 Hz, H-6b), 4.01–4.07 (m, 2H, H-4, H-5), 3.74 (dddd, 1H, $J_{F,5'}$ 25.4 Hz, $J_{5',4'}$ 10.1 Hz, $J_{5',6'a}$ 3.9 Hz, $J_{5',6'b}$ 1.7 Hz, H-5'), 3.63 (ddd, 1H, $J_{4',3'}$ 9.3 Hz, $J_{4',5'}$ 10.1 Hz, $J_{4',OH}$ 6.3 Hz, H-4'), 3.25 (d, 1H, $J_{4',OH}$ 6.3 Hz, 4'-OH), 2.19 (s, 3H, CH_3), 2.09 (s, 3H, CH_3), 2.07 (s, 3H, CH_3), 2.05 (s, 3H, CH_3), 1.99 (s, 3H, CH_3), 1.96 (s, 3H, CH_3). ^{13}C NMR (150.90 MHz, $CDCl_3$): δ 171.47 (C=O), 171.00 (C=O), 170.81 (C=O), 170.10 (C=O), 169.98 (C=O), 169.12 (C=O), 95.84 (C-1'), 88.97 (C-1), 81.66 (d, $J_{F,6'}$ 173.6 Hz, C-6'), 72.48 (C-3), 72.15 (C-3'), 72.09 ($J_{F,5'}$ 18.0 Hz, C-5'), 71.55 (C-4),70.27 (C-5), 70.07 (C-2'), 69.80 (C-

2), 68.12 (d, $J_{F,4'}$ 7.4 Hz, C-4'), 62.33 (C-6), 21.12–20.51 (CH_3). **β-anomer:** ^{19}F (564.69 MHz, $CDCl_3$): δ -235.65 (td, $J_{F,6'}$ 47.3 Hz, $J_{F,5}$ 25.1 Hz). ^{1}H NMR (600.13 MHz, $CDCl_3$): δ 5.73 (d, 1H, $J_{1,2}$ 8.2 Hz, H-1), 5.39 (d, 1H, $J_{1',2'}$ 4.0, H-1'), 5.29 (dd, 1H, $J_{3,2}$ 9.3 Hz, $J_{3,4}$ 8.8, H-3), 5.18 (dd, 1H, $J_{3',2'}$ 10.5 Hz, $J_{3',4'}$ 9.4 Hz, H-3'), 4.97 (dd, 1H, $J_{2,1}$ 8.2 Hz, $J_{2,3}$ 9.3 Hz, H-2), 4.80 (dd, 1H, $H_{2',1'}$ 4.0 Hz, $H_{2',3'}$ 10.5 Hz, H-2'), 4.64 (ddd, 1H, $J_{F,6'a}$ 47.3 Hz, $J_{6'a,5}$ 3.9 Hz, $J_{6'a,6'b}$ 10.3 Hz, H-6'a), 4.57 (ddd, 1H, $J_{F,6'}$ 47.3 Hz, $J_{6'b,5}$ 1.7 Hz, $J_{6'b,6'a}$ 10.3 Hz, H-6'b), 4.46 (dd, 1H, $J_{6a,5}$ 2.3 Hz, $J_{6a,6b}$ 12.3 Hz, H-6a), 4.18 (dd, 1H, $J_{6b,5}$ 4.0 Hz, $J_{6b,6a}$ 12.3 Hz, H-6b), 4.08 (dd, 1H, $J_{4,3}$ 8.8 Hz, $J_{4,5}$ 9.7 Hz, H-4), 3.81 (ddd, 1H, $J_{5,4}$ 9.7 Hz, $J_{5,6a}$ 2.3 Hz, $J_{5,6b}$ 4.0 Hz, H-5), 3.75 (dddd, 1H, $J_{F,5'}$ 25.1 Hz, $J_{5',4'}$ 10.1, $J_{5',6'a}$ 3.9 Hz, $J_{5',6'b}$ 1.7 Hz, H-5'), 3.65 (ddd, 1H, $J_{4',3'}$ 9.4 Hz, $J_{4',5'}$ 10.1 Hz, $J_{4',OH}$ 6.3 Hz, H-4'), 2.94 (d, 1H, $J_{OH,4'}$ 6.3 Hz, 4'-OH), 2.11 (s, 3H CH_3), 2.09 (s, 6H CH_3), 2.05 (s, 3H CH_3), 2.00 (s, 6H, CH_3). ^{13}C NMR (150.90 MHz, $CDCl_3$): δ 171.84 (C=O), 170.93 (C=O), 170.79 (C=O), 170.27 (C=O), 169.76 (C=O), 169.03 (C=O), 95.78 (C-1'), 91.39 (C-1), 81.66 (d, $J_{F,6'}$ 173.6 Hz, C-6'), 75.56 (C-3), 73.21 (C-5), 72.52 (C-3'), 72.12 (d, $J_{F,5'}$ 18.0 Hz, C-5'), 71.84 (C-4), 71.08 (C-2), 69.93 (C-2'), 68.38 (d, $J_{F,4'}$ 7.5 Hz, C-4'), 62.47 (C-6), 21.00-20.69 (CH_3). MS: Calcd for [$C_{24}H_{33}FO_{16}$]: *m/z* 596.51: ESIMS found: $[M + Na]^+$ 619.1.

4-*O*-(6'-deoxy-6'-fluoro-α-D-glucopyranosyl)-D-glucopyranoside (15):

According to the procedure for compound **6,** deprotection of the acetyl groups of compound **14** (14 mg, 0.023 mmol) yielded 6 mg (75%) of the 6'-F-maltose. **α-anomer:** ^{19}F (564.69 MHz, $CDCl_3$): δ -235.79 (td, $J_{F,6'}$ 47.3 Hz, $J_{F,5'}$ 28.1 Hz). ^{1}H NMR (600.13 MHz, $CDCl_3$): δ 5.44 (d, 1H, $J_{1',2'}$ 3.9 Hz, H-1'), 5.22 (d, 3.8 Hz, H-1), 4.73 (ddd, 1H, $J_{6'a,F}$ 47.3 Hz, $J_{5',6'a}$ 3.7 Hz, $J_{6'a,6'b}$ 10.7 Hz, H-6'a), 4.68 (ddd, 1H, $J_{6'b,F}$ 47.3 Hz, $J_{5',6'b}$ 1.8 Hz, $J_{6'a,6'b}$ 10.7 Hz, H-6'b), 3.96 (dd, 1H, $J_{3,2}$ 9.9 Hz, $J_{3,4}$ 9.1 Hz, H-3), 3.93 (ddd, 1H, $J_{5,4}$ 10.0 Hz, $J_{5,6a}$ 2.3 Hz, $J_{5,6b}$ 4.6 Hz , H-5), 3.91–

3.82 (m, 1H, H-5‘), 3.82 (dd, 1H, $J_{6a,5}$ 2.3 Hz, $J_{6a,6b}$ 12.2 Hz, H-6a), 3.77 (dd, 1H, $J_{6b,5}$ 4.6 Hz, $J_{6b,6a'}$ 12.2 Hz, $J_{6'a,}$ H-6b), 3.71 (dd, 1H, $J_{2',3'}$ 9.9 Hz, $J_{3',4'}$ 9.3 Hz, H-3‘), 3.65 (dd, 1H, $J_{4,3}$ 9.1 Hz, $J_{4,5}$ 10.0 Hz, H-4), 3.59 (dd, 1H, $J_{2',1'}$ 3.9 Hz, $J_{2',3'}$ 9.9 Hz, H-2‘), 3.56 (dd, 1H, $J_{2,1}$ 3.8 Hz, $J_{2,3}$ 9.9 Hz, H-2), 3.50 (dd, 1H, $J_{3',4'}$ 9.3 Hz, $J_{4',5'}$ 10.2 Hz, H-4’).^{13}C NMR (150.90 MHz, $CDCl_3$): δ 100.03 (C-1’), 92.24 (C-1), 82.54 (d, $J_{F,6'}$ 167.8 Hz, C-6’), 77.24 (C-4), 73.60 (C-3), 73.03 (C-3’), 71.99 (C-2’), 71.89 (C-2), 71.77 ($J_{F,5'}$ 17.4 Hz, C-5’), 70.19 (C-5), 68.63 (d, $J_{F,4'}$ 6.9 Hz, C-4’), 60.87 (C-6). **β-anomer:** ^{19}F (564.69 MHz, $CDCl_3$): δ -235.77 (td, $J_{F,6'}$ 47.3 Hz, $J_{F,5'}$ 28.0 Hz). ^{1}H NMR (600.13 MHz, $CDCl_3$): δ 5.44 (d, 1H, $J_{1',2'}$ 3.9 Hz, H-1’), 4.725 (ddd, 1H, $J_{6'a,F}$ 47.3 Hz, $J_{5',6'a}$ 3.7 Hz, $J_{6'a,6'b}$ 10.7 Hz, H-6’a), 4.68 (ddd, 1H, $J_{6'b,F}$ 47.3 Hz, $J_{5',6'b}$ 1.8 Hz, $J_{6'a,6'b}$ 10.7 Hz, H-6’b), 4.64 (d, 1H, $J_{1,2}$ 8.0 Hz, H-1), 3.91–3.82 (m, 1H, H-5‘), 3.88 (dd, 1H, $J_{6a,5}$ 2.1 Hz, $J_{6a,6b}$ 12.2 Hz, H-6a), 3.73 (dd, 1H, $J_{6b,5}$ 5.2 Hz, $J_{6a,6b}$ 12.2 Hz, H-6b), 3.76 (dd, 1H, $J_{3,2}$ 9.5 Hz, $J_{3,4}$ 9.1, H-3), 3.70 (dd, 1H, $J_{2',3'}$ 9.9 Hz, $J_{3',4'}$ 9.3 Hz, H-3‘), 3.65 (dd, 1H, $J_{4,3}$ 9.1 Hz, $J_{4,5}$ 9.8 Hz, H-4), 3.59 (dd, 1H, $J_{2',1'}$ 3.9 Hz, $J_{2',3'}$ 9.9 Hz, H-2‘), 3.70–3.60 (m, 1H, H-5), 3.50 (dd, 1H, $J_{3',4'}$ 9.3 Hz, $J_{4',5'}$ 10.2 Hz, H-4’), 3.26 (dd, 1H, $J_{2,1}$ 8.0 Hz, $J_{2,3}$ 9.5 Hz, H-2). ^{13}C NMR (150.90 MHz, $CDCl_3$): δ 99.92 (C-1’), 96.12 (C-1), 82.54 (d, $J_{F,6'}$ 167.8 Hz, C-6’), 77.02 (C-4), 76.57 (C-3), 74.80 (C-5), 74.35 (C-2), 73.01 (C-3’), 71.78 ($J_{F,5'}$ 17.4 Hz, C-5’), 71.64 (C-2’), 68.60 (d, $J_{F,4'}$ 6.8 Hz, C-4’), 60.99 (C-6).

1,2,3-Tri-*O*-acetyl-4-*O*-(2’,3’-di-*O*-acetyl-4’,6’-benzylidene-α-D-glucopyranosyl)-6-*O*-*tert*-butyldimethylsilyl-D-glucopyranoside (17):

To a cooled solution of 4’,6’-benzylidenemaltose **10** (579 mg, 1.38 mmol) and imidazol (219 mg, 1.61 mmol) in dry DMF (10 mL), *t*-butyldimethylsilyl chloride (218 mg, 1.61. mmol) was added and the reaction mixture was stirred for 24 h at room temperature. Then the solvents

were removed under vacuum, the residue was redissolved in dry pyridine (10 mL) and cooled to 0 °C, and then acetic anhydride (2.5 mL, 26 mmol) was added. After 12 h stirring at room temperature, the mixture was poured into ice/water (100 mL). The precipitate was removed by filtration and purified by column chromatography (PE/EE 3:2) to afford the product **17** as a colorless foam (437 mg, 43%) with an α/β = 1 : 0.8. **α-anomer**: ^{1}H NMR (600.13 MHz, $CDCl_3$): δ 7.44–7.41 (m, 2H, H phenyl), 7.36–7.34 (m, 3H, H phenyl), 6.25 (d, 1H, $J_{1,2}$ 3.7 Hz, H-1), 5.53 (dd, 1H, $J_{3,2}$ 10.2 Hz, $J_{3,4}$ 9.2 Hz, H-3), 5.48 (s, 1H, CH-phenyl), 5.47 (dd, 1H, $J_{3',2'}$ 10.2 Hz, $J_{3',4'}$ 9.6 Hz, H-3'), 5.39 (d, 1H, $J_{1',2'}$ 4.0 Hz, H-1'), 4.881 (dd, 1H, $J_{2,1}$ 3.7 Hz, $J_{2,3}$ 10.2 Hz, H-2), 4.879 (dd, 1H, $J_{2',1'}$ 4.0 Hz, $J_{2',3'}$ 10.2 Hz, H-2'), 4.32 (dd, 1H, $J_{6'a,5'}$ 4.8 Hz, $J_{6'a,6'b}$ 10.2 Hz, H-6'a), 4.15 (dd, 1H, $J_{4,3}$ 9.2 Hz, $J_{4,5}$, 9.2 Hz, H-4), 4.00–3.91 (m, 1H, 6a), 3.96–3.91 (m, 1H, H-5'), 3.87–3.85 (m, 2H, H-5, H-6b), 3.72 (dd, 1H, $J_{6'b,5'}$ 8.1, $J_{6'a,6'b}$ 10.2, H-6'b), 3.63 (dd, 1H, $J_{4',3'}$ 9.6 Hz, $J_{4',5'}$ 9.6 Hz, H-4'), 2.20–1.98 (5s, 15H, CH_3), 0.90 (s, 9H, $C(CH_3)_3$), 0.08 (s, 3H, Si-CH_3), 0.07 (s, 3H, Si- CH_3). ^{13}C NMR (150.90 MHz, $CDCl_3$): δ 170.89–169.16 (C=O), 137.03 (C-1 phenyl), 128.32 / 126.37 (C-2,3,5,6 phenyl), 101.76 (CH-phenyl), 96.41 (C-1'), 89.29 (C-1), 79.16 (C-4'), 73.22 (C-5), 72.50 (C-3), 70.84 (C-4), 70.17 (C-2), 68.82 (C-6'), 68.72 (C-3'), 63.76 (C-5'), 61.73 (C-6), 26.14 ($C(\underline{C}H_3)_3$), 21.23-20.63 (CH_3), 18.58 ($\underline{C}(CH_3)_3$), -4.86 (Si-CH_3). **β-anomer**: ^{1}H NMR (600.13 MHz, $CDCl_3$): δ 7.44–7.41 (m, 2H, H-phenyl), 7.36–7.34 (m, 3H, H-phenyl), 5.70 (d, 1H, $J_{1,2}$ 8.2 Hz, H-1), 5.47 (s, 1H, CH-phenyl), 5.45 (dd, 1H, $J_{3',2'}$ 10.2 Hz, $J_{3',4'}$ 9.6 Hz, H-3'), 5.39 (d, 1H, $J_{1',2'}$ 4.0 Hz, H-1'), 5.30 (dd, 1H, $J_{3,2}$ 9.4 Hz, $J_{3,4}$ 9.0, H-3), 4.91 (dd, 1H, $J_{2,1}$ 8.2 Hz, $J_{2,3}$ 9.4 Hz, H-2), 4.86 (dd, 1H, $J_{2',1'}$ 4.0 Hz, $J_{2',3'}$ 10.2 Hz, H-2'), 4.31 (dd, 1H, $J_{6'a,5'}$ 4.8 Hz, $J_{6'a,6'b}$ 10.2 Hz, H-6'a), 4.145 (dd, 1H, $J_{4,3}$ 9.9 Hz, $J_{4,5}$ 9.5 Hz, H-4), 4.00–3.91 (m, 1H, 6a,6b), 3.96–3.91 (m, 1H, H-5'), 3.70 (dd, 1H, $J_{6'b,5'}$ 8.1 Hz, $J_{6'a,6'b}$ 10.2 Hz, H-6'b), 3.61 (dd, 1H, $J_{4',3'}$ 9.6 Hz, $J_{4',5'}$ 9.6 Hz, H-4'), 3.56 (dt, 1H, $J_{5,4}$ 9.5 Hz, $J_{5,6}$ 2.3 Hz), 2.20–1.98 (5s,

15H, CH_3), 0.89 (s, 9H, $C(CH_3)_3$), 0.06 (s, 3H, Si-CH_3), 0.05 (s, 3H, Si-CH_3). ^{13}C NMR (150.90 MHz, $CDCl_3$): δ 170.89–169.16 (C=O), 137.04 (C-1 phenyl), 128.31 (C-2,3,5,6 phenyl), 129.19 (C-4 phenyl), 101.77 (CH-phenyl), 96.13 (C-1'), 91.56 (C-1), 79.17 (C-4'), 75.63 (C-3), 75.65 (C-5), 71.42 / 71.36 / 71.31 / 70.27 (C-2'α/β, C-2, C-4), 68.81 (C-6'), 68.70 (C-3'), 63.66 (C-5'), 61.70 (C-6), 26.16 ($\underline{C}(CH_3)_3$), 21.23-20.63 (CH_3), 18.66 ($C(\underline{C}H_3)_3$), -4.88 (Si-CH_3). MS: Calcd for [$C_{35}H_{50}O_{16}Si$]: *m/z* 754.85: ESIMS found: $[M + Na]^+$ 777.3.

1,2,3-Tri-*O*-acetyl-4-*O*-(2',3'-di-*O*-acetyl-4',6'-benzylidene-α-D-glucopyranosyl)-6-deoxy-6-fluoro-D-glucopyranoside (18):

Deprotection of the silyl group and subsequent fluorination was performed in a one-pot procedure using Deoxofluor. The TBDMS protected maltose derivative **17** (190 mg, 0.25 mmol) was heated under reflux with 50% Deoxofluor solution (in toluene, 1.1 g, 2.5 mmol) and dry CH_2Cl_2 (5 mL) for 48 h. The solution was cooled to room temperature, quenched with methanol (2 mL) neutralized with conc. ammonia (pH~7) and concentrated under vacuum. The residue was chromatographed on silica gel (PE/EE 3:2) to obtain product **18** as a colorless foam (27 mg, 17%). ^{19}F (564.69 MHz, $CDCl_3$): δ -237.43 (ddd, $J_{F,6a}$ 47.1 Hz, $J_{F,6b}$ 48.0 Hz, $J_{F,5}$ 30.2 Hz), ^{1}H NMR (400.13 MHz, $CDCl_3$): δ 7.45–7.43 (m, 2H, H-phenyl), 7.37–7.34 (m, 3H, H-phenyl), 6.28 (d, 1H, $J_{1,2}$ 3.7 Hz, H-1), 5.56 (ddd, 1H, $J_{3,2}$ 10.1 Hz, $J_{3,4}$ 9.3 Hz, $J_{3,F}$ 0.8 Hz, H-3), 5.50 (s, 1H, CH-phenyl), 5.48 (dd, 1H, $J_{3',2'}$ 10.2 Hz, $J_{3',4'}$ 9.6 Hz, H-3'), 5.43 (d, 1H, $J_{1',2'}$ 4.2 Hz, H-1'), 4.97 (dd, 1H, $J_{2,1}$ 3.7 Hz, $J_{2,3}$ 10.1 Hz, H-2), 4.90 (dd, 1H, $J_{2',1'}$ 4.2 Hz, $J_{2',3'}$ 10.2 Hz, H-2'), 4.79 (ddd, 1H, $J_{F,6a}$ 47.1 Hz, $J_{6a,5}$ 2.2 Hz, $J_{6a,6b}$ 10.9 Hz, H-6a), 4.63 (ddd, 1H, $J_{F,6b}$ 48.0 Hz, $J_{6b,5}$ 1.0 Hz,

$J_{6a,6b}$ 10.9 Hz, H-6b), 4.31 (dd, 1H, $J_{6'a,5'}$ 94.3Hz, $J_{6'a,6'b}$ 9.8 Hz, H-6'a), 4.21 (dd, 1H, $J_{4,3}$ 9.3 Hz, $J_{4,5}$ 9.9 Hz, H-4), 4.03 (dddd, 1H, $J_{F,5}$ 30.2 Hz, $J_{5,4}$ 9.9 Hz, $J_{5,6a}$ 2.2 Hz, $J_{5,6b}$ 1.0 Hz, H-5), 3.81 (ddd, $J_{5',4'}$ 9.8 Hz, $J_{5',6'a}$ 4.3 Hz, $J_{5',6'b}$ 9.6 Hz, H-5'), 3.74 (t, $J_{6'b,5'}$ 9.8 Hz, $J_{6'b,6'a}$ 9.8 Hz, H-6'b), 3.66 (t, 1H, $J_{4',3'}$ 9.6 Hz, $J_{4',5'}$ 9.6 Hz, H-4'), 2.21 (s, 3H, CH_3), 2.09 (s, 3H, CH_3), 2.07 (s, 3H, CH_3), 2.03 (s, 3H, CH_3), 1.99 (s,3H, CH_3). ^{13}C NMR (100.61 MHz, $CDCl_3$): δ 170.92 (C=O), 170.18 (C=O), 169.97 (C=O), 169.88 (C=O), 169.07 (C=O), 136.87 (C-1 phenyl), 128.38 / 126.22 (C-2,3,5,6 phenyl), 129.24 (C-4 phenyl), 101.53 (CH-phenyl), 96.57 (C-1'), 89.21 (C-1), 81.36 (d, $J_{F.6}$ 176.8 Hz, C-6), 78.75 (C-4'), 72.52 (C-3), 71.44 (d, $J_{F,5}$ 18.3 Hz, C-5), 71.01 (C-2'), 70.05 (d, $J_{F,4}$ 7.6 Hz, C-4), 69.98 (C-2), 68.72 (C-3'), 68.56 (C-6'), 64.00 (C-5'), 21.13 (CH_3), 21.09 (CH_3), 20.92 (CH_3), 20.73 (CH_3), 20.55 (CH_3).

4-*O*-(α-D-glucopyranosyl)-6-deoxy-6-fluoro-D-glucopyranoside (20):

Deprotection was performed by heating compound **18** (13 mg, 0.02 mmol) under reflux with conc. acetic acid (2 mL) for ½ h. The reaction mixture was poured into water (2 mL) and extracted with ethyl acetate (3 x 5 mL). The combined organic layers were washed with 10% aqueous $NaHCO_3$ and brine (5 mL), dried over $MgSO_4$, concentrated under reduced pressure and chromatographed over silica (PE/EE 2:7) to yield product **19** (8 mg, 73%). Deprotection of the acetyl groups (8 mg, 0.014 mmol) with sodium methoxide (0.4 equiv.) according to the procedure reported for compound **6** afforded 6-deoxy-6-fluoro maltose **20** (2 mg, 40%). **α-anomer:** ^{19}F (564.69 MHz, D_2O): δ -234.88 (m). ^{1}H NMR (600.13 MHz, D_2O): δ 5.46 (d, 1H, $J_{1',2'}$ 3.9 Hz, H-1'), 5.24 (d, 1H, $J_{1,2}$ 3.8 Hz, H-1), 4.74 (ddd, 1H, $J_{6a,F}$ 47.0 Hz, $J_{6a,5}$ 3.0 Hz, $J_{6a,6b}$ 10.7 Hz, H-6^{a}), 4.64 (ddd, 1H, $J_{6b,F}$ 48.1 Hz, $J_{6b,5}$ 1.7 Hz, $J_{6a,6b}$ 10.7 Hz, H-6b), 4.11–4.03 (m, 1H, H-5), 3.98 (dd, 1H, $J_{3,2}$ 9.8 Hz, $J_{3,4}$ 8.9 Hz, H-3), 3.85 (dd, 1H, $J_{6'a,5'}$ 2.2 Hz, $J_{6'a,6'b}$ 12.4 Hz, H-

6’a), 3.77–3.67 (m, 3H, H-6’b, H-4, H-5’), 3.69 (dd, 1H, $J_{3',2'}$ 9.9 Hz, $J_{3',4'}$ 9.3 Hz, H-3’), 3.575 (dd, 1H, $J_{2',1'}$ 3.9 Hz, $J_{2',3'}$ 9.9 Hz, H-2’), 3.57 (dd, 1H, $J_{2,1}$ 3.8 Hz, $J_{2,3}$ 9.8 Hz, H-2), 3.41 (dd, 1H, $J_{4',3'}$ 9.3 Hz, $J_{4',5'}$ 9.9 Hz, H-4’). ^{13}C NMR (150.90 MHz, D_2O): δ 99.77 (C-1’), 92.39 (C-1), 82.76 (d, $J_{F,6}$ 168.2 Hz, C-6), 75.47 (d, $J_{F,4}$ 6.4 Hz, C-4), 73.56 (C-3), 73.14–73.10 (C-3’,5’), 72.02 (C-2’), 71.60 (C-2), 69.68 (C-4’), 69.14 (d, $J_{F,5}$ 17.3 Hz, C-5), 60.83 (C-6). **β-anomer:** ^{19}F (564.69 MHz, D_2O): δ -235.41 (t, $J_{F,6}$ 47.5 Hz,, $J_{F,5'}$ 30.7 Hz). ^{1}H NMR (600.13 MHz, D_2O): δ 5.457 (d, 1H, $J_{1',2'}$ 3.9 Hz, H-1’), 4.80 (ddd, 1H, $J_{6a,F}$ 47.2 Hz, $J_{6a,5}$ 2.9 Hz, $J_{6a,6b}$ 10.7 Hz, H-6a), 4.70 (ddd, 1H, $J_{6b,F}$ 47.9 Hz, $J_{6b,5}$ 1.7 Hz, $J_{6a,6b}$ 10.7 Hz, H-6b), 4.69 (d, 1H, $J_{1,2}$ 8.1 Hz, H-1), 3.84 (dd, 1H, $J_{6'a,5'}$ 2.3 Hz, $J_{6'a,6'b}$ 12.4 Hz, H-6a), 3.79 (dd, 1H, $J_{3,2}$ 9.0 Hz, $J_{3,4}$ 8.0 Hz, H-3), 3.77–3.67 (m, 4H, H-6’b, H-4, H-5, H-5’), 3.68 (dd, 1H, $J_{3',2'}$ 10.0 Hz, $J_{3',4'}$ 9.3, H-3’), 3.576 (dd, 1H, $J_{2',1'}$ 3.9 Hz, $J_{2',3'}$ 10.0 Hz, H-2’), 3.41 (dd, 1H, $J_{4',3'}$ 9.3 Hz, $J_{4',5'}$ 9.9 Hz, H-4’), 3.28 (dd, 1H, $J_{2,1}$ 8.1 Hz, $J_{2,3}$ 9.0 Hz, H-2). ^{13}C NMR (150.90 MHz, D_2O): δ 99.70 (C-1’), 96.27 (C-1), 82.50 (d, $J_{F,6}$ 168.6 Hz, C-6), 76.45 (C-3), 75.26 (d, $J_{F,4}$ 6.6 Hz, C-4), 74.27 (C-2), 73.43 (d, $J_{F,5}$ 17.8 Hz, C-5), 73.14–73.10 (C-3’,5’), 71.94 (C-2’), 69.65 (C-4’), 60.83 (C-6).

1,2,3,6-Tetra-*O*-acetyl-4-*O*-(2’,3’-di-*O*-acetyl-α-D-glucopyranosyl)-D-glucopyranoside (21): Maltose derivative **10** was peracetylated under standard conditions. The benzylidene group was then selectively cleaved, starting from 868 mg (1.27 mmol) of derivative **11** and according to the procedure described for compound **20**. The crude mixture was purified by chromatography over silica (PE/EE 1:5) to yield product **21** (576 mg, 76%) with a α/β ratio of 5:2. **α-anomer:** ^{1}H NMR (600.13 MHz, $CDCl_3$): δ 6.24 (d, 1H, $J_{1,2}$ 3.7 Hz, H-1), 5.51 (dd, 1H, $J_{3,2}$ 10.2 Hz, $J_{3,4}$ 8.8 Hz, H-3), 5.37 (d, 1H, $J_{1',2'}$ 4.0 Hz, H-1’), 5.23 (dd, 1H, $J_{3',2'}$ 10.6 Hz, $J_{3',4'}$ 8.8 Hz, H-3’), 4.97 (dd, 1H, $J_{2,1}$ 3.7 Hz, $J_{2,3}$ 10.2 Hz, H-2), 4.78 (dd, 1H, $J_{2',1'}$ 4.0 Hz, $J_{2',3'}$ 10.6 Hz, H-2’), 4.47 (dd,

1H, $J_{6a,5}$ 2.3 Hz, $J_{6a,6b}$ 12.4 Hz, H-6a), 4.22 (dd, 1H, $J_{6b,5}$ 3.7 Hz, $J_{6a,6b}$ 12.4 Hz, H-6b), 4.10 (ddd, 1H, $J_{5,4}$ 10.0 Hz, $J_{5,6a}$ 2.3 Hz, $J_{5,6b}$ 3.7 Hz, H-5), 4.04 (dd, 1H, $J_{4,3}$ 8.8 Hz, $J_{4,5}$ 10.0 Hz, H-4), 3.84–3.81 (m, 2H, H-6'a, H-6'b), 3.71–3.65 (m, 2H, H-4', H-5'), 2.93 (d, 1H, $J_{4',OH}$ 4.9 Hz, 4'-OH), 2.22 (s, 3H, CH_3), 2.14 (s, 3H, CH_3), 2.10 (s, 3H, CH_3), 2.08 (s, 3H, CH_3), 2.02 (s, 3H, CH_3), 1.99 (s, 3H, CH_3). ^{13}C NMR (100.61 MHz, $CDCl_3$): δ 171.56 (C=O), 171.22 (C=O), 170.95 (C=O), 170.10 (C=O), 170.04 (C=O), 169.14 (C=O), 96.07 (C-1'), 89.01 (C-1), 72.76 (C-5'), 72.53 (C-3), 72.19 (C-3'), 72.00 (C-4), 70.42 (C-5), 70.28 (C-2'), 70.09 C-4'), 69.44 (C-3'), 69.83 (C-2), 62.74 (C-6), 62.45 (C-6'), 21.21–20.60 (CH_3). **β-anomer:** ^{1}H NMR (60013 MHz, $CDCl_3$): δ 5.74 (d, 1H, $J_{1,2}$ 8.2 Hz, H-1), 5.35 (d, 1H, $J_{1',2'}$ 4.0 Hz, H-1'), 5.29 (dd, 1H, $J_{3,2}$ 9.3 Hz, $J_{3,4}$ 8.8 Hz, H-3), 5.19 (dd, 1H, $J_{3',2'}$ 10.6 Hz, $J_{3',4'}$ 9.1 Hz, H-3'), 4.98 (dd, 1H, $J_{2,1}$ 8.2 Hz, $J_{2,3}$ 9.3 Hz, H-2), 4.77 (dd, 1H, $J_{2',1'}$ 4.0 Hz, $J_{2',3'}$ 10.6 Hz, H-2'), 4.49 (dd, 1H, $J_{6a,5}$ 2.4 Hz, $J_{6a,6b}$ 12.4 Hz, H-6a), 4.21 (dd, 1H, $J_{6b,5}$ 4.4 Hz, $J_{6a,6b}$ 12.4 Hz, H-6b), 4.03 (dd, 1H, $J_{4,3}$ 8.8 Hz, $J_{4,5}$ 9.7 Hz, H-4), 3.84–3.78 (m, 3H, H-5, H-6'a, H-6'b), 3.71–3.65 (m, 2H, H-4', H-5'), 2.96 (d, 1H, $J_{4',OH}$ 4.9 Hz, 4'-OH), 2.13 (s, 3H, CH_3), 2.09 (s, 3H, CH_3), 2.10 (s, 3H, CH_3), 2.05 (s, 3H, CH_3), 2.012 (s, 3H, CH_3), 2.01 (s, 3H, CH_3). ^{13}C NMR (100.61 MHz, $CDCl_3$): δ 171.63 (C=O), 171.23 (C=O), 170.91 (C=O), 170.24 (C=O), 170.04 (C=O), 168.99 (C=O), 95.96 (C-1'), 91.40 (C-1), 75.53 (C-3), 73.30 (C-5), 72.78 (C-5'), 72.23 (C-3'), 72.12 (C-4), 71.08 (C-2), 70.20 (C-2'), 70.13 (C-4'), 62.82 (C-6), 62.42 (C-6'), 21.21–20.60 (CH_3). MS: Calcd for [$C_{24}H_{34}O_{17}$]: *m/z* 594.52: ESIMS found: $[M + Na]^+$ 617.6.

1,2,3,6-Tetra-*O*-acetyl-4-*O*-(2',4'-di-*O*-acetyl-6'-deoxy-6'-fluoro-α-D-galactopyranosyl)-D-glucopyranoside (22):

According to the general procedure for compound **13**, fluorination of the 6'-hydroxy group was promoted by microwave irradiation, starting from 170 mg (0.29 mmol) of derivative **21**. The crude mixture was purified by flash column chromatography (silica gel, PE/EE 1:2) to give 50 mg (30%) of compound **22**. **α-anomer:** ^{19}F (564.69 MHz, $CDCl_3$): δ -230.88 (td, $J_{F,6'}$ 46.8 Hz, $J_{F,5'}$ 15.1 Hz). ^{1}H NMR (600.13 MHz, $CDCl_3$): δ 6.24 (d, 1H, $J_{1,2}$ 3.7 Hz, H-1), 5.51 (dd, 1H, $J_{3,2}$ 10.2 Hz, $J_{3,4}$ 8.7 Hz, H-3), 5.47 (d, 1H, $J_{1',2'}$ 4.0 Hz, H-1'), 5.39 (dd, 1H, $J_{4',3'}$ 3.4 Hz, $J_{4',5'}$ 1.4 Hz, H-4'), 5.00 (dd, 1H, $J_{2',1'}$ 4.0 Hz, $J_{2',3'}$ 10.7 Hz, H-2'), 4.98 (dd, 1H, $H_{2,1}$ 3.7 Hz, $H_{2,3}$ 10.2 Hz, H-2), 4.48 (dd, 1H, $J_{6a,5}$ 2.1 Hz, $J_{6a,6b}$ 12.4 Hz, H-6a), 4.41 (ddd, 1H, $J_{F,6'a}$ 46.2 Hz, $J_{6'a,5'}$ 4.3 Hz, $J_{6'a,6'b}$ 9.9 Hz, H-6'a), 4.38 (ddd, 1H, $J_{F,6'b}$ 47.4 Hz, $J_{6'b,5'}$ 6.6 Hz, $J_{6'b,6'a}$ 69.9 Hz, H-6'b), 4.15 (dd, 1H, $J_{3',2'}$ 10.7 Hz, $J_{3',4'}$ 3.4 Hz, H-3'), 4.17–4.12 (m, 1H, H-5'), 4.10 (dd, 1H, $J_{6b,5}$ 3.5 Hz, $J_{6a,6b}$ 12.4 Hz, H-6b), 4.08 (dd, 1H, $J_{4,3}$ 8.7 Hz, $J_{4,5}$ 10.0 Hz, H-4), 4.04 (ddd, 1H, $J_{5,4}$ 9.9 Hz, $J_{5,6a}$ 2.1 Hz, $J_{5,6b}$ 3.5 Hz, H-5), 2.19–1.99 (CH_3). ^{13}C NMR (150.90 MHz, $CDCl_3$): δ 171.57 (C=O), 170.99 (2 C=O), 170.17 (C=O), 169.96 (C=O), 169.04 (C=O), 96.27 (C-1'), 89.05 (C-1), 81.78 (d, $J_{F,6'}$ 172.5 Hz, C-6'), 72.43 (C-3), 71.70 (C-4), 70.52 (C-5), 70.48 (C-2'), 70.39 (d, $J_{F,4'}$ 6.1 Hz, C-4'), 69.80 (C-2), 68.94 (d, $J_{F,5'}$ 21.6 Hz, C-5'), 65.99 (C-3'), 62.27 (C-6), 21.18–20.59 (CH_3). **β-anomer:** ^{19}F (564.69 MHz, $CDCl_3$): δ -230.81 (td, $J_{F,6'}$ 46.8 Hz, $J_{F,5'}$ 15.3 Hz). ^{1}H NMR (600.13 MHz, $CDCl_3$): δ 5.73 (d, 1H, $J_{1,2}$ 8.2 Hz, H-1), 5.45 (d, 1H, $J_{1',2'}$ 4.0 Hz, H-1'), 5.39 (dd, 1H, $J_{4',3'}$ 3.5 Hz, $J_{4',5'}$ 1.4 Hz, H-4'), 5.28 (dd, 1H, $J_{3,2}$ 9.5 Hz, $J_{3,4}$ 8.8 Hz, H-3), 4.99 (dd, 1H, $J_{2',1'}$ 4.0 Hz, $J_{2',3'}$ 10.7 Hz, H-2'), 4.98 (dd, 1H, $H_{2,1}$ 8.2 Hz, $H_{2,3}$ 9.5 Hz, H-2), 4.50 (dd, 1H, $J_{6a,5}$ 2.3 Hz, $J_{6a,6b}$ 12.3 Hz, H-6a), 4.42 (ddd, 1H, $J_{F,6'a}$ 46.2 Hz, $J_{6'a,5'}$ 4.3 Hz, $J_{6'a,6'b}$ 9.9 Hz, H-6'a), 4.38 (ddd, 1H, $J_{F,6'b}$ 47.4 Hz, $J_{6'b,5'}$ 6.7 Hz, $J_{6'b,6'a}$ 9.9 Hz, H-6'b), 4.14 (dd, 1H, $J_{3',2'}$ 10.7 Hz, $J_{3',4'}$ 3.5 Hz, H-3'), 4.18–4.11 (m, 1H, H-5'), 4.09 (dd, 1H $J_{6b,5}$ 4.4 Hz, $J_{6b,6a}$ 12.3 Hz, H-6b), 4.07 (dd, 1H, $J_{4,3}$ 8.8 Hz, $J_{4,5}$ 9.7 Hz, H-4), 3.77 (ddd, 1H, $J_{5,4}$ 9.7 Hz, $J_{5,6a}$ 2.2 Hz, $J_{5,6b}$ 4.4 Hz,

H-5), 2.14–2.01 (CH_3). ^{13}C NMR (150.90 MHz, $CDCl_3$): δ 171.59 (C=O), 170.97 (C=O), 170.96 (C=O), 170.27 (C=O), 169.73 (C=O), 169.04 (C=O), 96.13 (C-1'), 91.41 (C-1), 81.83 (d, $J_{F,6'}$ 172.6 Hz, C-6'), 75.59 (C-3), 73.43 (C-5), 71.73 (C-4), 71.06 (C-2), 70.49 (C-2'), 70.40 (d, $J_{F,4'}$ 6.2 Hz, C-4'), 68.95 (d, $J_{F,5'}$ 21.5 Hz, C-5'), 66.05 (C-3'), 62.42 (C-6), 21.05-20.69 (CH_3).

4-*O*-(6'-deoxy-6'-fluoro-α-D-galactopyranosyl)-D-glucopyranoside (23):

According to the general procedure for compound **6**, deprotection of the acetyl groups of compound **22** (37 mg, 0.062 mmol) afforded derivative **23** in 99% (21 mg).

α-anomer (β>α): ^{19}F (564.69 MHz, D_2O): δ -230.42 (ddd, $J_{F,6'a}$ 45.4 Hz, $J_{F,6'b}$ 48.1 Hz, $J_{F,5'}$ 16.4 Hz) ^{1}H NMR (600.13 MHz, D_2O): δ 5.412 (d, 1H, $J_{1',2'}$ 3.0 Hz, H-1'), 5.22 (d, 1H, $J_{1,2}$ 3.8 Hz, H-1α), 4.66 (ddd, 1H, $J_{F,6'a}$ 45.4 Hz, $J_{5',6'a}$ 3.5 Hz, $J_{6'a,6'b}$ 10.1 Hz, H-6'a), 4.60 (ddd, 1H, $J_{F,6'b}$ 48.1 Hz, $J_{5',6'b}$ 7.7 Hz, $J_{6'a,6'b}$ 10.1 Hz, H-6'b), 4.31–4.25 (m, 1H, H-5'), 4.04–4.03 (m, 1H, H-4'), 3.95 (dd, 1H, $J_{3,2}$ 10.0 Hz, $J_{3,4}$ 8.9 Hz, H-3), 3.90–3.85 (m, 3H, H-5, H-2', H-3'), 3.85 (dd, 1H, $J_{6a,5}$ 2.4 Hz, $J_{6a,6b}$ 12.2 Hz, H-6a), 3.78 (dd, 1H, $J_{6b,5}$ 5.1 Hz, $J_{6a,6b}$ 12.2 Hz, H-6b), 3.60 (dd, 1H, $J_{4,3}$ 8.9 Hz, $J_{4,5}$ 10.0 Hz, H-4), 3.57 (dd, 1H, $J_{2,1}$ 3.8 Hz, $J_{2,3}$ 10.0 Hz, H-2). ^{13}C NMR (150.90 MHz, D_2O): δ 100.70 (C-1'), 92.22 (C-1), 83.92 (d, $J_{F,6'}$ 165.4 Hz, C-6'), 78.24 (C-4), 73.61 (C-3), 71.52 (C-2), 70.46 (d, $J_{F,5'}$ 19.7 Hz, C-5'), 70.35 (C-5), 69.39 (d, $J_{F,3'}$ 1.7 Hz, C-3'), 69.22 (d, $J_{F,4'}$ 7.6 Hz, C-4'), 69.92 (C-2'), 61.01 (C-6). **β-anomer:** ^{19}F (564.69 MHz, $CDCl_3$): δ -230.41 (ddd, $J_{F,6'a}$ 45.4 Hz, $J_{F,6'b}$ 48.1 Hz, $J_{F,5'}$ 16.4 Hz). ^{1}H NMR (600.13 MHz, D_2O): δ 5.416 (d, 1H, $J_{1',2'}$ 3.0 Hz, H-1'), 4.66 (ddd, 1H, $J_{F,6'a}$ 45.4 Hz, $J_{5',6'a}$ 3.5 Hz, $J_{6'a,6'b}$ 10.1 Hz, H-6'a), 4.64 (d, 1H, $J_{1,2}$ 8.0 Hz, H-1), 4.60 (ddd, 1H, $J_{F,6'b}$ 48.1 Hz, $J_{5',6'b}$ 7.7 Hz, $J_{6'a,6'b}$ 10.1 Hz, H-6'b), 4.31–4.25 (m, 1H, H-5'), 4.04–4.03 (m, 1H, H-4'), 3.90 (dd, 1H, $J_{6a,5}$ 2.1 Hz, $J_{6a,6b}$ 12.3 Hz, H-6a), 3.88–3.85 (m, 2H, H-2', H-3'), 3.74 (dd, 1H, $J_{6b,5}$ 5.1 Hz, $J_{6a,6b}$ 12.3 Hz, H-6b), 3.76–3.73 (m, 1H, H-

3), 3.60 (dd, 1H, $J_{4,3}$ 8.9 Hz, $J_{4,5}$ 10.0 Hz, H-4), 3.60–3.56 (m, 1H, H-5), 3.27 (dd, 1H, $J_{2,1}$ 8.0 Hz, $J_{2,3}$ 9.6 Hz, H-2). ^{13}C NMR (150.90 MHz, D_2O): δ 100.59 (C-1'), 96.12 (C-1), 83.92 (d, $J_{F,6'}$ 165.4 Hz, C-6'), 77.99 (C-4), 76.58 (C-3), 74.94 (C-5), 74.22 (C-2), 70.48 (d, $J_{F,5'}$ 19.7 Hz, C-5'), 69.38 (d, $J_{F,3'}$ 1.7 Hz, C-3'), 69.20 (d, $J_{F,4'}$ 7.7 Hz, C-4'), 68.82 (C-2'), 61.16 (C-6).
